# High‐Polarity Fluoroalkyl Ether Electrolyte Enables Solvation‐Free Li^+^ Transfer for High‐Rate Lithium Metal Batteries

**DOI:** 10.1002/advs.202104699

**Published:** 2021-12-19

**Authors:** Liwei Dong, Yuanpeng Liu, Kechun Wen, Dongjiang Chen, Dewei Rao, Jipeng Liu, Botao Yuan, Yunfa Dong, Ze Wu, Yifang Liang, Mengqiu Yang, Jianyi Ma, Chunhui Yang, Chuan Xia, Baoyu Xia, Jiecai Han, Gongming Wang, Zaiping Guo, Weidong He

**Affiliations:** ^1^ National Key Laboratory of Science and Technology on Advanced Composites in Special Environments and Center for Composite Materials and Structures Harbin Institute of Technology Harbin 150080 China; ^2^ MIIT Key Laboratory of Critical Materials Technology for New Energy Conversion and Storage School of Chemistry and Chemical Engineering Harbin Institute of Technology Harbin 150080 China; ^3^ State Key Laboratory of Urban Water Resource and Environment Harbin Institute of Technology Harbin 150080 China; ^4^ School of Mechanical Engineering Chengdu University Chengdu 610106 China; ^5^ School of Material Science and Engineering Jiangsu University Zhenjiang Jiangsu 212013 China; ^6^ Institute of Atomic and Molecular Physics Sichuan University Chengdu Sichuan 610065 China; ^7^ School of Materials and Energy University of Electronic Science and Technology of China Chengdu 611731 China; ^8^ Key Laboratory of Material Chemistry for Energy Conversion and Storage (Ministry of Education) Hubei Key Laboratory of Material Chemistry and Service Failure Wuhan National Laboratory for Optoelectronics School of Chemistry and Chemical Engineering Huazhong University of Science and Technology (HUST) 1037 Luoyu Road Wuhan 430074 China; ^9^ Department of Chemistry and Hefei National Laboratory for Physical Science at Microscale University of Science and Technology of China Anhui 230026 China; ^10^ School of Chemical Engineering and Advanced Materials The University of Adelaide Adelaide SA 5005 Australia

**Keywords:** fluoroalkyl ether, high rate, lithium metal batteries, Li^+^ solvation structure, long cycle

## Abstract

Lithium metal batteries (LMBs) have aroused extensive interest in the field of energy storage owing to the ultrahigh anode capacity. However, strong solvation of Li^+^ and slow interfacial ion transfer associated with conventional electrolytes limit their long‐cycle and high‐rate capabilities. Herein an electrolyte system based on fluoroalkyl ether 2,2,2‐trifluoroethyl‐1,1,2,3,3,3‐hexafluoropropyl ether (THE) and ether electrolytes is designed to effectively upgrade the long‐cycle and high‐rate performances of LMBs. THE owns large adsorption energy with ether‐based solvents, thus reducing Li^+^ interaction and solvation in ether electrolytes. With THE rich in fluoroalkyl groups adjacent to oxygen atoms, the electrolyte owns ultrahigh polarity, enabling solvation‐free Li^+^ transfer with a substantially decreased energy barrier and ten times enhancement in Li^+^ transference at the electrolyte/anode interface. In addition, the uniform adsorption of fluorine‐rich THE on the anode and subsequent LiF formation suppress dendrite formation and stabilize the solid electrolyte interphase layer. With the electrolyte, the lithium metal battery with a LiFePO_4_ cathode delivers unprecedented cyclic performances with only 0.0012% capacity loss per cycle over 5000 cycles at 10 C. Such enhancement is consistently observed for LMBs with other mainstream electrodes including LiCoO_2_ and LiNi_0.5_Mn_0.3_Co_0.2_O_2_, suggesting the generality of the electrolyte design for battery applications.

## Graphical abstract

This work designs an electrolyte system based on fluoroalkyl ether 2,2,2‐trifluoroethyl‐1,1,2,3,3,3‐hexafluoropropyl ether (THE) to effectively upgrade the long‐cycle and high‐rate performances of lithium metal batteries. With THE rich in fluoroalkyl groups adjacent to oxygen atoms, the electrolyte owns ultrahigh polarity and enables solvation‐free Li^+^ transfer with a substantially decreased energy barrier.

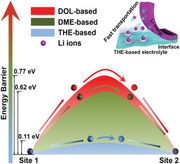

## 1 Introduction

Lithium metal batteries (LMBs) have been regarded as promising future energy storage devices to meet the requirements of high‐capacity, long cycle, and high rate energy storage.^[^ ^1^, ^2^, ^3^, ^4^, ^5^, ^6^ ^]^ There still exist unsatisfactory and even disastrous drawbacks associated with current LMBs, including degrading capacities over cycles, low rate capacities, and safety issues due to the high reactivity of the Li metal anode, all of which are largely attributed to the larger energy barrier of Li^+^ solvation and the small ionic conductivity at the Li anode/electrolyte interface with conventional ether and carbonate electrolytes.^[^ ^7^, ^8^, ^9^ ^]^ In addition, these commercial ether‐based and carbonate‐based electrolytes are highly flammable, posting potential safety hazards.^[^ ^10^, ^11^ ^]^ Extensive efforts have been focused on developing alternative electrolyte systems to address these challenges including: 1) solid‐state electrolytes with safety and high‐energy‐density, such as inorganic solid electrolytes^[^ ^12^ ^]^ and solid‐state polymer electrolytes;^[^ ^13^ ^]^ 2) electrolyte solvents with excellent physicochemical properties, such as ionic liquid with excellent ion conductivity,^[^ ^14^ ^]^ nontoxic and nonflammable aqueous solvents,^[^ ^15^ ^]^ and nitrile sovlents with a high oxidation potential;^[^ ^16^ ^]^ 3) electrolyte additives with solid electrolyte interface (SEI) forming and flame‐retardant capabilities, such as lithium difluorobis (oxalato) phosphate (LiDFBOP),^[^ ^17^ ^]^ fumed silica,^[^ ^18^ ^]^ trimethyl phosphate,^[^ ^19^ ^]^ and acetic acid (HAc);^[^ ^20^ ^]^ 4) high concentrated electrolytes with advanced solvation structures;^[^ ^21^, ^22^ ^]^ 5) precycling step to regulate the coordination between solvent and salt.^[^ ^23^ ^]^ However, solid‐state electrolytes tend to form rough electrolyte/Li interface and induce large interfacial impedance and low ionic conductivity, and liquid electrolytes for LMBs still face challenges to ensure fast Li^+^ transfer while ensuring proper viscosity and physicochemical compatibility with separators and electrodes.

In this report, we develop an electrolyte system based on fluoroalkyl ether 2,2,2‐trifluoroethyl‐1,1,2,3,3,3‐hexafluoropropyl ether (THE) and ether electrolytes to address the aforementioned fundamental issues associated with the electrolytes for LMBs. The THE electrolyte with electron‐withdrawing fluoroalkyl groups adjacent to oxygen atoms suppresses Li^+^‐solvation and enables efficient LiF‐formation, thus significantly enhancing in‐cell ionic conduction of LMBs, and effectively mitigating Li dendrite growth. With the electrolyte, the LiFePO_4_ cathode exhibits unprecedented cyclic performances with 0.0012% capacity loss cycle^−1^ over 5000 cycles at 10 C, in addition to the upgraded performances of LMBs with LiCoO_2_ (LCO) and LiNi_0.5_Co_0.2_Mn_0.3_O_2_ (NCM523). The mechanism of “solvation‐free Li^+^ transfer” is first proposed to elucidate the high‐rate performances of an electrolyte with an intermediate ionic conductivity. Our work provides valuable insights into the molecular structure design of fluorinated ethers for the applications in energy storage in general.

## 2 Results and Discussion

### 2.1 Physicochemical Properties of THE Electrolyte

**Figure 1:**
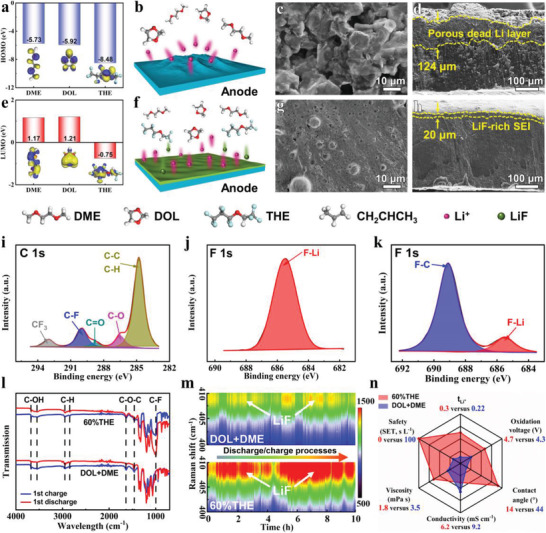
a) HOMO and e) LUMO values of DME, DOL, and THE. Schematics of anode surface and Li^+^ transmission in b) DOL + DME and f) 60%THE electrolytes. Titled SEM and cross‐sectional SEM images of Li anodes in c,d) DOL + DME and g,h) 60%THE electrolytes after 100 cycles, yellow lines show the etching depth. XPS of the SEI layers in i,j) 60%THE and k) DOL + DME electrolytes, C 1s and F 1s are presented, including peak deconvolution and assignments. l) FTIR spectra of Li metal in DOL + DME and 60%THE electrolytes. m) In situ Raman images of DOL + DME and 60%THE electrolytes in discharge/charge. n) Comparison of the properties of conventional DOL + DME and 60%THE electrolytes.

**Figure 2:**
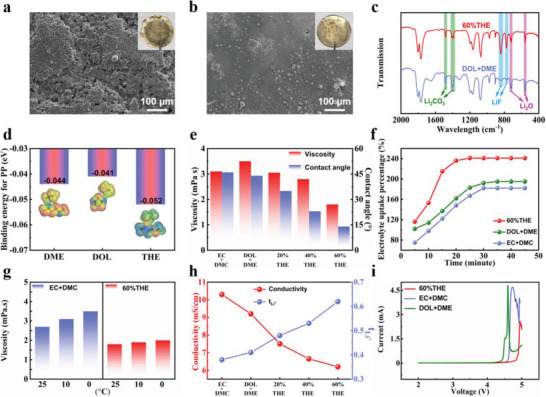
SEM images of the Li metals in a) DOL + DME and b) 60%THE electrolytes after 100 cycles. c) FTIR spectrum of the SEI layers in 60%THE and DOL + DME electrolytes. d) Adsorption energy and corresponding electron density maps for CH_3_CH_2_CH_3_ with DME, DOL, and THE. e) Viscosity for various electrolytes, and contact angle between different electrolytes and PP separator. f) Uptake curves of PP separator for different electrolytes over time. g) Viscosity of EC + DMC and 60%THE electrolytes at different temperatures. h) Conductivity and $t_{Li^{+}}$ with different electrolytes. i) Electrochemical windows of EC + DMC, DOL + DME, and 60%THE electrolytes.

**Figure 3:**
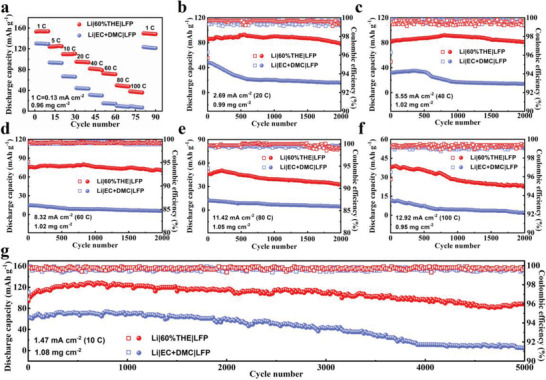
a) Rate performances of the Li/LFP cells with EC + DMC and 60%THE electrolytes. Cycling performances of the Li/LFP cells with EC + DMC and 60%THE electrolytes at b) 20 C, c) 40 C, d) 60 C, e) 80 C, f) 100 C, and g) 10 C.

The compositions of the electrolytes investigated in this work are shown in Table S1 of the Supporting Information. THE has outstanding antioxidation stability owing to robust electron‐withdrawing groups (CF_3_ and CF_2_). As shown in **Figure** **1**a,e, it exhibits a much lower highest occupied molecular orbital (HOMO) energy value (−8.48 eV) and a much lower lowest unoccupied molecular orbitals (LUMO) level (−0.75 eV) as compared with commercial 1, 3‐dioxolane (DOL) and dimethoxyether (DME) solvents, indicating its high‐voltage stability and ready reduction on the anode with SEI formation. The negative LUMO value of THE is stemmed from the excellent electron affinity of high‐polarity CF_3_ and CF_2_ groups. As shown in Figure 1b, conventional DOL + DME electrolyte causes uneven deposition of Li^+^ since rough and sluggish interfacial ion transference between anode and electrolyte produces dead Li on the anode surface, leading to irreversible Li^+^ transmission. After 100 cycles in the DOL + DME electrolyte, a loose layer is observed on the surface of the Li anode (**Figure** **2**a). This porous layer (Figure 1c) further aggravates the rough deposition, resulting in continuous corrosion of the Li metal, as evidenced with the low Coulombic efficiency (CE) and poor cycling stability (**Figure** **3**b–g). The porous dead Li layer with a thickness of 124 µm is clearly observed from the cross‐sectional scanning electron microscopy (SEM) image (Figure 1d). This means that the reversibility and uniformity of Li^+^ deposition morphology are inferior in the DOL + DME electrolyte, causing the rapid decay of the capacity.^[^ ^24^ ^]^ By contrast, for 60%THE electrolyte, abundant LiF is formed at the electrolyte/electrode interface (Figure 1f). LiF owns excellent ion transmission ability and stability (6.4 V vs Li/Li^+^),^[^ ^25^ ^]^ and enables compact packing in the SEI to isolate the Li metal from the electrolyte. Furthermore, LiF owns high interfacial energy with Li metal,^[^ ^26^ ^]^ which accelerates Li^+^ migration at the interface and promotes the parallel growth of Li dendrites on Li metal plane instead of vertical growth. The protective effect of LiF‐rich SEI on the Li anode is clearly observed in the SEM images. The anode surface layer in the 60%THE electrolyte (Figures 1g and 2b) is much more compactly integrated than that in the DOL + DME electrolyte. Especially, the corrosion of the Li metal is greatly reduced in the 60%THE electrolyte, and only the top layer (20 µm) is corroded after 100 cycles (Figure 1h). The dense surface layer owns three advantages: 1) high robustness because the ball‐like Li morphology is less likely to pierce the separator; 2) large CE because the dense layer prevents continuous reaction to reduce the consumption of the Li anode and the electrolyte; and 3) additional volumetric capacity because dense Li packing reduces the volume. In the elemental mappings of Li anodes in DOL + DME (Figure S1a,b, Supporting Information) and 60%THE (Figure S1c,d, Supporting Information) electrolytes after cycling, elemental mapping of C is mainly derived from electrolyte solvents and, thus selected as the representative of organic species.^[^ ^27^ ^]^ The Li metal in the 60%THE electrolyte contains less C element as compared with that in the DOL + DME electrolyte, implying reduced solvent decomposition in the 60%THE electrolyte. Elemental mapping of F is taken as the representative of the SEI layer.^[^ ^27^ ^]^ The Li metal in the 60%THE electrolyte contains more F element as compared with that in the DOL + DME electrolyte, indicating that the addition of THE is favorable for the formation of the SEI layer. Figure 1i–k and Figure S2 (Supporting Information) show the X‐ray photoelectron spectroscopy (XPS) of the SEI layers in 60%THE and DOL + DME electrolytes. The organic species formed with the ether electrolyte solvent, including C═O, C—O, and C—H/C—C, are studied with C 1s spectrum (Figure 1i). Obvious signals of CF_3_ and C–F are observed, as attributed to the cleavage of the fluorinated groups of THE. As shown in Figure 1j, a main peak at ≈685.7 eV is observed, implying that the F element in the SEI layer exists mainly in the form of the F—Li bond.^[^ ^28^ ^]^ For the SEI layer in the DOL + DME electrolyte, significant F–C and weak F–Li signals are observed (Figure 1k). In addition, the Fourier transform infrared (FTIR) spectrum of the Li anodes with 60%THE and DOL + DME electrolytes after cycling is taken (Figure 2c). For the Li anode with the 60%THE electrolyte, the signals of LiF,^[^ ^29^ ^]^ Li_2_CO_3_,^[^ ^30^ ^]^ and Li_2_O^[^ ^30^ ^]^ are observed. For the Li anode with the DOL + DME electrolyte, the LiF signal is significantly weaker than that of the Li anode with the 60%THE electrolyte, which is consistent with the XPS results (Figure 1j,k). To understand the effect of THE electrolyte on LiF formation at high temperatures, we conduct XPS analysis on Li anodes disassembled from the cells with different electrolytes after 50 cycles at 60 °C, as shown in Figure S3 of the Supporting Information. For the 60%THE electrolyte, obvious F–Li signal is observed, indicating that LiF formation is facilitated in the 60%THE electrolyte at 60 °C. As shown in Figure 1l, FTIR is used to study the internal change of THE‐based electrolyte during charging and discharging. For the 60%THE electrolyte, the peak of fluorine‐containing species (C–F stretching vibration) at ≈1000 cm^−1^ increases apparently after the first charge,^[^ ^31^ ^]^ indicating the cleavage of the fluorinated segment of THE molecules. By contrast, there is no obvious fluorine‐containing signal in the DOL + DME electrolyte. This is also demonstrated through in situ Raman spectroscopy. The test battery configuration is shown in Figure S4 of the Supporting Information. As displayed in Figure 1m, extensive LiF (≈409 cm^−1^) is formed in the 60%THE electrolyte, considerably above that in the DOL + DME electrolyte, which is attributed to the cleavage of C–F bond in THE.

Flammable commercial electrolytes exhibit safety risk in high‐rate operations of lithium metal batteries.^[^ ^32^ ^]^ The thermal stability of ethylene carbonate (EC) + dimethyl carbonate (DMC), DOL + DME, and 60%THE electrolytes is evaluated, as shown in Figure S5 and Videos S1–S3 of the Supporting Information. In Videos S1–S3 of the Supporting Information, commercial EC + DMC and DOL + DME electrolytes are readily ignited and burned quickly, whereas the 60% THE electrolyte is nonflammable even with repeated igniting. While exposed to fire, there are raging flames in EC + DMC and DOL + DME electrolytes, but no flame is observed in the 60%THE electrolyte (Figure S5c, Supporting Information). The THE solvent without flash point is incombustible. In addition, THE, as a highly fluorinated ether‐based solvent with abundant fluoroalkyl groups, owns the highest binding energy with oxygen radical among these solvents, as displayed in Figure S6 of the Supporting Information. Therefore, the noncombustible THE effectively inhibits the propagation of free oxygen radicals during combustion, decreasing the flammability of the electrolyte, which is consistent with the previous research on fluoroalkyl ethers.^[^ ^10^, ^28^, ^33^ ^]^ Rigorous thermal runaway tests are conducted by measuring differential scanning calorimetry and open‐circuit voltage (OCV) curves.^[^ ^34^ ^]^ As shown in Figure S7a of the Supporting Information, the 60%THE electrolyte exhibits an exothermic peak at 139 °C, significantly higher than those of DOL + DME (51 °C) and EC + DMC (59 °C) electrolytes, indicating better thermal stability of the 60%THE electrolyte. In addition, for the LMB with LiFePO_4_ (LFP) cathode and 60%THE electrolyte, a fall in the OCV takes place at 224 °C, considerably above those using DOL + DME (168 °C) and EC + DMC (186 °C) electrolytes (Figure S7b, Supporting Information). As shown in the insets in Figure S7b of the Supporting Information, the cells with the two commercial electrolytes broke after heating to 230 °C, while the cell with 60%THE electrolyte is intact without explosion signs, suggesting that the THE‐based electrolyte improves battery safety. Low concentrated lithium bis(trifluoromethane sulfonyl) imide (LiTFSI)‐based electrolytes tend to cause aluminum(Al) corrosion in 4 V class cathodes. Al current collector is oxidized to form Al^3+^, which is coordinated with solvent molecules and diffuses in the electrolyte to cause corrosion of the Al current collector.^[^ ^35^ ^]^ As displayed in Figure S8 of the Supporting Information, the SEM images show the morphology of the Al current collector in different electrolytes. The Al current collector in the commercial ether electrolyte experiences more severe corrosion than that in the 60%THE electrolyte. This is attributed to the lower binding energy of THE‐Al^3+^ (−0.67 eV) compared to those of DOL‐AL^3+^ (−0.92 eV) and DME‐Al^3+^ (−2.03 eV), causing difficult coordination between Al^3+^ and THE solvent (Figure S9, Supporting Information). Therefore, the solubility of Al^3+^ in the 60%THE electrolyte is reduced, which alleviates the Al corrosion. The affinity of electrolyte solvents with the polypropylene (PP) separator is studied with density functional theories (DFT). As shown in Figure 2d, the adsorption energy of THE‐CH_3_CH_2_CH_3_ (−0.052 eV) is much larger than those of DOL‐CH_3_CH_2_CH_3_ (−0.041 eV) and DME‐CH_3_CH_2_CH_3_ (−0.044 eV), indicating the outstanding affinity between PP and THE. The 60%THE electrolyte owns a high wettability with PP separator and various electrodes (Figure 2e,f; Figure S10, Supporting Information), reducing the interphase impedance and enhancing the utilization of active materials. The viscosity of the 60%THE electrolyte is only 1.8 mPa s, considerably lower than those of EC + DMC (3.1 mPa s) and DOL + DME (3.5 mPa s) electrolytes. In particular, the viscosity of the 60%THE electrolyte maintains nearly unchanged at low temperatures, as compared with EC + DMC and DOL + DME electrolytes (Figure 2g; Figure S11, Supporting Information). The conductivities and Li^+^ transference numbers ($t_{Li^{+}}$) of the electrolytes are displayed in Figure 2h and Figures S12 and S13 (Supporting Information). The conductivity of the 60%THE electrolyte is slightly lower, but reasonably comparable to those of EC + DMC and DOL + DME electrolytes (that is, 6.2 vs 10.3 and 9.2 mS cm^−1^). Different from conductivity, $t_{Li^{+}}$ increases with increasing THE in the electrolytes from 0.48 for 20%THE, and 0.53 for 40%THE to 0.62 for 60%THE, which are all above those for EC + DMC (0.38) and DOL + DME (0.41). In particular, as shown in Figure 2i, compared with commercial electrolytes, the 60%THE electrolyte owns a wider electrochemical window and is, thus, capable of supporting higher voltage battery systems. As shown in Figure S14 of the Supporting Information, the nucleation overpotential of the 60%THE electrolyte is 104 mV, considerably lower than that of the EC + DMC electrolyte (167 mV). The lower nucleation overpotential indicates that the SEI layer in the 60%THE electrolyte facilitates uniform Li nucleation and allows for rapid Li^+^ transfer. As displayed in Figure S15 of the Supporting Information, the cycling of the symmetrical Li/Li cell with the 60%THE electrolyte maintains stable for 150 h with a low over potential (≈6.4 mV). By contrast, the cell with the EC+DMC electrolyte fails to achieve a stable performance for more than 75 h.

### 2.2 Electrochemical Behaviors of Commercial Electrodes with THE Electrolyte

As displayed in Figure S16 of the Supporting Information, the redox peaks of the 60%THE electrolyte own higher intensities at ≈3.25 and ≈3.60 V than those of the EC + DMC electrolyte, indicating that the 60%THE electrolyte with solvation‐free Li^+^ transfer significantly improves the electrochemical reaction kinetics. As shown in Figure S17 of the Supporting Information, the Li/LFP battery with the 60%THE electrolyte owns the largest capacity and the electrochemical performances of the 60%THE electrolyte are investigated. Due to the high ion transmission capability of LiF‐rich SEI and excellent separator wettability, the 60%THE electrolyte greatly increases the rate and cyclic performances of the battery. As shown in Figure 3a, the LFP cathode with the 60%THE electrolyte delivers discharge capacities from 153.2 to 38.1 mAh g^−1^ as the rate increases from 1 C to 100 C. The capacity recovers to 97.8% of the initial capacity as the current density is decreased from 100 C back to 1 C, indicating the excellent reversibility of the 60%THE electrolyte. By contrast, the discharge capacities are considerably lower at all C rates for the EC + DMC electrolyte. As displayed in Figure S18 of the Supporting Information, the overpotentials at all rates of the Li/LFP cell with the 60%THE electrolyte are considerably lower than those of the Li/LFP cell with the EC + DMC electrolyte, indicating the significantly reduced polarization in the 60%THE electrolyte. As displayed in Figures S19 and S20 of the Supporting Information, the Li/LFP cells with the 60%THE electrolyte also exhibit better electrochemical performances than the EC + DMC electrolyte at low rates. A high‐loading LFP electrode of 13.46 mg cm^−2^ is employed. As shown in Figure S21 of the Supporting Information, the battery with the 60%THE electrolyte maintains a stable cycle at 0.61 mA cm^−2^, while the battery with the EC + DMC electrolyte undergoes decrease in both capacity and CE. As shown in Figure S22 of the Supporting Information, the electrochemical impedance analysis shows that the semicircle diameter at the high frequency of the 60%THE electrolyte is smaller than that with commercial electrolyte, indicating that the formed electrolyte/electrode interface owns a smaller charge transfer resistance. As displayed in Figure S23 of the Supporting Information, after 50 cycles, the impedance of the Li/LFP cell with the 60%THE electrolyte increases slightly, while that of the EC + DMC electrolyte increases significantly after 50 cycles. The Li/LTO battery using 60%THE electrolyte also exhibits obvious advantages in electrochemical performances over the commercial electrolyte (Figure S24, Supporting Information). In addition, the LFP cathode is assembled into full cells with LTO and graphite anodes. Significantly improved cycling and rate performances are achieved in full batteries (Figures S25 and S26, Supporting Information). For other major commercial cathode materials including LCO and NCM532, the batteries with the 60%THE electrolyte also deliver improved rate and cycling performances as compared with those with the commercial electrolyte (Figures S27 and S28, Supporting Information), indicating that the 60%THE electrolyte owns a wide‐range applicability. It is noted that less improvement in performances is observed for NCM811 full cells using the 60%THE electrolyte (Figure S29, Supporting Information), as attributed to the cation‐mixing due to the similarity in radius between Li^+^ and Ni^2+^.^[^ ^36^ ^]^ Due to the extremely low HOMO energy value (<−8.4 eV) and the high oxidation potential (>5.6 V), THE is free of oxidation on the surface of the cathode (Figure 1a; Figure S30, Supporting Information). Therefore, the composition of cathode electrolyte interphase in the 60%THE electrolyte is the same as that in commercial electrolyte and, thus, the 60%THE electrolyte cannot effectively inhibit cation‐mixing.^[^ ^37^ ^]^ The layered structure of the nickel‐rich cathode gradually evolves into an electrochemically inert rock salt phase, resulting in the rapid deterioration of the electrochemical performances of the nickel‐rich material.

The long‐cycle, high‐rate performances of the Li/LFP cells with EC + DMC and 60%THE electrolytes are shown in Figure 3b–g of the Supporting Information. The cells with the 60%THE electrolyte own larger capacities and better capacity retention as compared with the EC + DMC electrolyte at all rates (Table S2, Supporting Information). In particular, the battery shows unprecedented cycle retention with only 0.0012% capacity loss per cycle over 5000 cycles at 10 C (Figure S31a and Table S3, Supporting Information), which is the lowest capacity loss for the Li/LFP cells as reported to this date. Moreover, the LFP cathode with the 60%THE electrolyte exhibits the highest rate capability among reported electrolyte engineering work for the Li/LFP cells (Figure S31b and Table S4, Supporting Information). To further demonstrate the superiority of the 60%THE electrolyte, FEC and VC, the most widely used SEI forming additives in commercial applications, are compared with THE. As shown in Figure S32 of the Supporting Information, the Li/LFP cell with the 60%THE electrolyte delivers a discharge capacity of 119.8 mAh g^−1^ after 1375 cycles at 10 C, which is considerably larger than those with EC + DMC + 10%FEC + 2%VC (78.3 mAh g^−1^) and EC + DMC (56.6 mAh g^−1^) electrolytes. Therefore, FEC and VC additives have less pronounced impact on the high‐rate performances of the Li/LFP battery as compared with THE. The low‐temperature performances of the Li/LFP batteries with EC + DMC and 60%THE electrolytes are studied (Figure S33, Supporting Information). The battery with the 60%THE electrolyte also exhibits more pronounced rate capacities as compared with the commercial electrolyte at low temperatures. As displayed in Table S5 of the Supporting Information, with the 60%THE electrolyte, the battery capacities have no significant change at various rates (<5 mAh g^−1^) at 25 and 0 °C. The temperature window of the THE electrolyte for efficient battery operation is evaluated by measuring battery cycling performance at different temperatures. As shown in Figure S34 of the Supporting Information, the Li/LFP cell with the 60%THE electrolyte shows excellent cycling stability in a wide temperature range from −50 °C to +100 °C. Owing to the excellent thermal stability of the THE (Figures S5 and S7, Supporting Information), the high temperature performance of the cell with the 60%THE electrolyte is also enhanced. On the other hand, the outstanding low‐temperature performances of the 60%THE electrolyte are due to its almost unchanged viscosity at various temperatures (Figure 2g) and excellent compatibility between electrolyte and PP separator (Figure 2d–f), ensuring efficient in‐cell ionic conduction. By contrast, the high viscosity of the EC + DMC electrolyte at low temperatures causes slow ion transport and the battery is severely polarized, leading to the low capacity. As shown in Figure S35 of the Supporting Information, the Li/LFP cell assembled with 60%THE electrolyte and PVDF separator delivers a high specific capacity of 142.3 mAh g^−1^ with 97.7% capacity retention after 50 cycles, demonstrating that the 60%THE electrolyte is compatible with PVDF separator.

### 2.3 Mechanism of Rate‐Performance Improvement with THE‐Based Electrolyte

**Figure 4:**
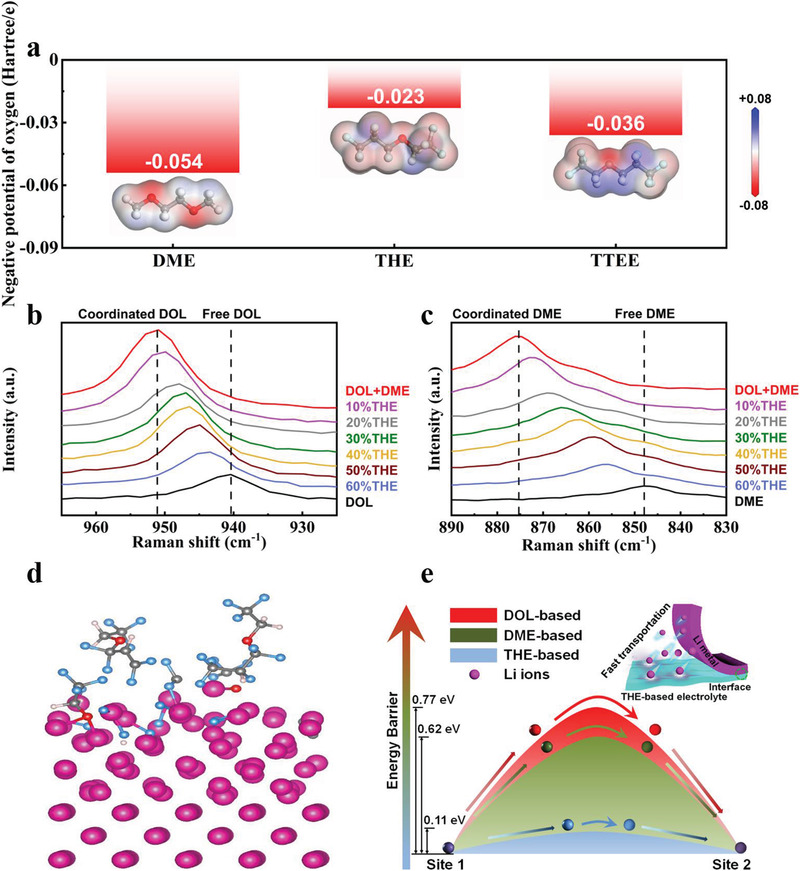
a) Oxygen negative potential and ESP comparisons of DME, THE, and TTEE. b,c) Raman spectra of different electrolytes (DOL + DME, 10%THE, 20%THE, 30%THE, 40%THE, 50%THE, and 60%THE electrolytes, the Li salt concentration is 1 m in these electrolytes) and electrolyte solvents (pure DOL and DME solvents without LiTFSI). d) Adsorption of fluorine‐rich THE on deposited Li (0 0 1) surface accompanied with abundant LiF formation through AIMD simulation. e) Energy barriers for Li transfer in DOL, DME, and THE solvents to deposited Li (0 0 1) surface through AIMD simulation.

Ab initio molecular dynamics (AIMD) simulations are employed to investigate the solvation structure and rate performances of electrolytes. Figure S36a–c of the Supporting Information shows the simulation snapshots of EC + DMC, DOL + DME, and 60%THE electrolytes, respectively. The representative configurations of coordinated molecules in the first Li^+^ shell in the three different electrolyte systems are depicted with a ball‐and‐stick model (Figure S36d–f, Supporting Information). Li^+^ prefers to coordinate with oxygen from EC, DMC, DOL, and DME solvent molecules, facilitating the dissociation of the lithium salts, while THE is a free solvent molecule and does not coordinate with either Li^+^ or anions. Therefore, the dissociation of lithium salts and the number of charge carriers in the THE‐based electrolyte decrease with increasing volume ratio of THE, resulting in reduced Li^+^ cations ($n_{Li^{+}}$) and TFSI^−^ anions ($n_{\mathrm{TFS}I^{-}}$). In the 60%THE electrolyte, the Li^+^ cations are weakly solvated with solvent molecules, and in the meanwhile the anions are seriously dragged by Li^+^ cations in return, resulting in low the mobility of TFSI^−^ anions ($\mu_{\mathrm{TFS}I^{-}}$). As shown in Equations (S1) and (S2), the ion conductivity and Li^+^ transference number own opposite trends with increasing volume ratio of THE (Figure 2h). As shown in Figure S37 of the Supporting Information, although the free solvent THE does not contribute to LiTFSI dissolution or Li^+^ solvation, the large dielectric constants of the DOL (7.1) and DME (7.2) in the 60%THE electrolyte still allow for the sufficient solvation of the lithium salt for battery operation at high rates. Electrostatic potential (ESP) is calculated to study the impact of electron‐withdrawing fluoroalkyl groups on the properties of solvent molecules (**Figure** **4**a). For the DME molecule, the negative potential (−0.054 Hartree e^−1^) mainly is concentrated on O atoms, while the O atom of the THE molecule owns a uniform negative potential (−0.023 Hartree e^−1^) distribution in the presence of electron‐withdrawing fluoroalkyl groups, demonstrating that THE is unable to coordinate with positively‐charged Li^+^. As the two F atoms on the carbon adjacent to the O atom are replaced with H atoms, the negative potential of the O atom of 2,2,2‐trifluoroethyl‐2,3,3,3‐tetrafluoropropyl ether (TTEE) is enhanced to −0.036 Hartree e^−1^, indicating that the two electron‐withdrawing fluoroalkyl groups adjacent to the oxygen atom result in the low solvating capability of lithium ions. Base on DFT calculation of the binding energy between DME and additive solvents, the intermolecular binding energy between THE and DME is calculated to be −0.144 eV, which is significantly higher than that between DME and the other commercial additives, such as FEC, VC, HFE, etc. (Figure S38a, Supporting Information). Moreover, the binding energy of THE–DOL is also the highest among the additive solvents (Figure S38b, Supporting Information). These results indicate that the solvent competition induced with THE addition weakens the interaction between Li^+^ and ether‐based solvents, which is further confirmed by Raman spectra and molecular dynamic (MD) simulation results (Figure 4b,c; Figure S39, Supporting Information). It is known that, after Li salt dissolving in solvent, the Raman peaks corresponding to the solvent undergo an obvious shift owing to the coordination between Li^+^ and solvent molecules.^[^ ^38^, ^39^ ^]^ As shown in Figure 4b,c, the Raman spectra of the DME/DOL undergoes a significant blue shift after coordination with Li ion. With the addition of THE, the peak shifts back, indicating the THE weakens the Li^+^ solvation structure. As displayed in Figure S39 of the Supporting Information, the MD results show that the coordination number decreases from 4.01 to 2.67 for DME‐Li^+^, from 0.45 to 0.26 for DOL‐Li^+^, upon THE addition. Moreover, the coordination number of THE with Li^+^ is 0, further confirming that THE does not participate the solvation structure of Li^+^, but reduces the coordination interaction between Li^+^ and ether‐based solvents, which leads to more efficient desolvation process of Li^+^ in the 60%THE electrolyte as compared to that in the DOL + DME electrolyte.

As shown in Tables S6 and S7 of the Supporting Information, the ion conductivity of each component in the battery with DOL + DME and 60%THE electrolytes is studied. The slowest ion conduction inside the battery occurs at the electrolyte/anode interface and is on the order of 10^−7^ S cm^−1^, which is far below that of the electrolytes. The ion transport at electrolyte/electrode interface and in the electrodes is contributed by Li^+^ transfer. Therefore, the Li^+^ conduction at the electrolyte/anode interface, instead of the Li^+^ conduction in the electrolyte, is the limiting factor for the rate performances of the battery system. With a smaller ionic conductivity, the THE‐based electrolyte owns a larger Li^+^ transference number and enables much enhanced rate performances of the batteries, indicating the THE‐based electrolyte greatly enhances the Li^+^ conduction at the electrolyte/anode interface. As shown in XPS (Figure 1j) and AIMD (Figure 4d) results, compared with DOL and DME molecules, abundant LiF forms at the surface of Li metal due to the C–F bond cleavage in THE, resulting in an increased F element ratio in the SEI layer. As shown in Figures 1j and 4d, the added F element ratio in the SEI layer exists almost entirely in the form of LiF after the introduction of THE. After introducing THE, the increased ratio of F element in SEI layer is ≈0.29 based on XPS results. Based on Equation (S3), *σ*_2_ is calculated to be ≈1.9 × 10^−7^ S cm^−1^, which is on the same order of that for LiF (*σ*_LiF_,6.4 × 10^−7^ S cm^−1^ − 1) and far beyond that of conventional electrolyte/anode interface (*σ*_1_,1 × 10^−9^ S cm^−1^) in the DOL + DME electrolyte.^[^ ^25^, ^40^ ^]^ As shown in Figure S40 of the Supporting Information, the Li^+^ diffusion barrier in LiF (0 0 1) is 0.19 eV,^[^ ^41^ ^]^ a value considerably lower than those in Li_2_CO_3_ (0 1 0) (0.28 eV) and Li_2_O (1 1 1) (0.45 eV),^[^ ^42^, ^43^ ^]^ which further indicates the lower transmission resistance of Li^+^ in LiF as compared with conventional electrolyte/anode interface. The enhancement on the ionic conductivity at electrolyte/anode interface gives rise to the improved rate performances with the 60%THE electrolyte.

The mechanism of C—F bond cleavage is further studied. Owing to a low LUMO energy value (Figure 1e), THE on the anode surface is prone to defluorination through reduction reaction. AIMD simulations (Figure S41, Supporting Information) show that the C—F bond breaks on the CF_2_ and the CF_3_ groups of THE. As shown in Figure S42 of the Supporting Information, the breaking energy values for the C—F bond cleavage of TTEE and 2,2,2‐trifluoroethyl‐1,1,2‐trifluoropropyl ether are 0.69 and 0.14 eV, considerably above that of THE (0.04 eV), indicating that the efficient formation of LiF through C—F bond cleavage with the THE electrolyte.

The energy barrier for Li^+^ mobility at the deposited Li (0 0 1)/electrolyte interface is calculated through AIMD simulations with a slow‐growth method (Figure 4e; Figure S43, Supporting Information). For the THE‐based electrolyte, the energy barrier is only on the order of ≈1/7 and 1/6 of those for the conventional DME and DOL electrolytes. We then speculate that Li^+^ does not completely strip off free‐solvent THE molecules during intercalation into the anode, verifying that the addition of THE is beneficial for improving the ion transmission at electrolyte/anode interface. The activation energy for Li^+^ transfer in different electrolytes is studied by measuring the interfacial resistance at various temperatures. Since ion transport in EC + DMC, DOL + DME, and 60%THE electrolytes is dependent on the mobility of the solvated molecules, the Li^+^ conductivity in these liquid electrolytes at different temperatures can be well described through the Vogel–Tammann–Fulcher empirical equation,^[^ ^44^ ^]^ as shown in Figure S44 of the Supporting Information. The activation energy barrier of the 60%THE electrolyte is 0.039 eV, considerably lower than those of EC + DMC (0.165 eV) and DOL + DME (0.219 eV) electrolytes, indicating that the Li^+^ transference in the 60%THE electrolyte is energetically more favorable. This is well consistent with the results of AIMD simulations. In addition, AIMD calculations (Figure 4d) show that F transfers from the THE solvent to the deposited Li surface to form LiF‐rich inorganic species near the anode surface, as observed with in situ Raman (Figure 1m). The LiF‐rich interphase layer on the anode improves the reaction kinetics and cyclic stability of the batteries.

## 3 Conclusion

In summary, THE‐based electrolyte is first developed by employing fluoroalkyl ether THE as a cosolvent of LiTFSI/DOL + DME. Both experiments and computational simulations demonstrate that the THE‐based electrolyte with interface ion affinity owns excellent wettability to the commercial separator, enables rapid and uniform Li^+^ transmission, and promotes the formation of LiF‐rich SEI with mitigated Li dendrites at the electrolyte/Li interface. In addition, the solvent competition induced with THE addition weakens the Li^+^ solvation structure of the electrolyte. Using the 60%THE electrolyte, the lithium metal battery with LiFePO_4_ cathode exhibits substantially enhanced cycling stability and favorable capacity fading rate. This study offers a promising strategy to endow THE‐based electrolytes with rapid interface ion transference for high‐performance energy‐storage devices.

## Conflict of Interest

The authors declare no conflict of interest.

## Supporting information

Supporting Information

Supplementary Video 1

Supplementary Video 2

Supplementary Video 3

## Data Availability

The data that support the findings of this study are available from the corresponding author upon reasonable request.
