# Supplementary material for: High‐Polarity Fluoroalkyl Ether Electrolyte Enables Solvation‐Free Li+ Transfer for High‐Rate Lithium Metal Batteries
Source: Adv Sci (Weinh). 2021 Dec 19;9(5):2104699. doi: 10.1002/advs.202104699 (PMC8844499; doi:10.1002/advs.202104699)
Supplement: Supplementary file 1 — Supporting Information [file ADVS-9-2104699-s001.pdf]

© 2020 Wiley-VCH GmbH

ADVANCED SCIENCE
Open Access

Supporting Information

for *Adv. Sci.,* DOI: 10.1002/advs.202104699

High-polarity fluoroalkyl ether electrolyte enables solvation-free Li^+ transfer for high-rate lithium metal batteries

Liwei Dong, Yuanpeng Liu, Kechun Wen, Dongjiang Chen, Dewei Rao, Jipeng Liu, Botao Yuan, Yunfa Dong, Ze Wu, Yifang Liang, Mengqiu Yang, Jianyi Ma, Chunhui Yang, Chuan Xia, Baoyu Xia, Jiecai Han, Gongming Wang, Zaiping Guo*, and Weidong He**

Supplementary Information

High-polarity fluoroalkyl ether electrolyte enables solvation-free Li^+ transfer for high-rate lithium metal batteries

Liwei Dong, Yuanpeng Liu, Kechun Wen, Dongjiang Chen, Dewei Rao, Jipeng Liu, Botao Yuan, Yunfa Dong, Ze Wu, Yifang Liang, Mengqiu Yang, Jianyi Ma, Chunhui Yang, Chuan Xia, Baoyu Xia, Jiecai Han, Gongming Wang, Zaiping Guo*, Weidong He**

Dr. L. Dong, Dr. Y. Liu, Dr. K. Wen, Dr. J. Liu, Dr. B. Yuan, Dr. Y. Dong, Dr. Y. Liang, Dr. M. Yang, Prof. J. Han, Prof. W. He

National Key Laboratory of Science and Technology on Advanced Composites in Special Environments, and Center for Composite Materials and Structures, Harbin Institute of Technology, Harbin 150080, China.

E-mail: weidong.he@hit.edu.cn

Dr. L. Dong, Dr. J. Liu, Dr. Z. Wu, Dr. Y. Liang, Dr. M. Yang, Prof. C. Yang

MIIT Key Laboratory of Critical Materials Technology for New Energy Conversion and Storage, School of Chemistry and Chemical Engineering, Harbin Institute of Technology, Harbin 150080, China.

Dr. L. Dong, Prof. C. Yang

State Key Laboratory of Urban Water Resource and Environment, Harbin Institute of Technology, Harbin 150080, China.

Dr. D. Chen, Prof. W. He

School of Physics, University of Electronic Science and Technology of China, Chengdu 611731, China.

E-mail: weidong.he@hit.edu.cn

Prof. D. Rao

School of Material Science and Engineering, Jiangsu University, Zhenjiang, Jiangsu 212013, China.

Prof. J. Ma

Institute of Atomic and Molecular Physics, Sichuan University, Chengdu, Sichuan 610065, China.

Prof. C. Xia

School of Materials and Energy, University of Electronic Science and Technology of China, Chengdu 611731, China.

Prof. B. Xia

Key Laboratory of Material Chemistry for Energy Conversion and Storage (Ministry of Education), Hubei Key Laboratory of Material Chemistry and Service Failure, Wuhan National Laboratory for Optoelectronics, School of Chemistry and Chemical Engineering, Huazhong University of Science and Technology (HUST), 1037 Luoyu Road, Wuhan, 430074, China.

Prof. G. Wang

Department of Chemistry and Hefei National Laboratory for Physical Science at Microscale, University of Science & Technology of China, Anhui 230026, China.
E-mail: wanggm@ustc.edu.cn
Prof. Z. Guo
School of Chemical Engineering & Advanced Materials, The University of Adelaide, Adelaide, SA 5005, Australia.
E-mail: zaiping.guo@adelaide.edu.au

Experimental section

Preparation of THE-based electrolytes. The LiTFSI was dried under vacuum at 60 °C for 12 h. A mixed solvent of DOL/DME (v/v = 1:1) was prepared. THE-based electrolytes were composed of 1 M LiTFSI dissolved in THE/DOL/DME. More specifically, 1 M LiTFSI was dissolved in a mixture of DOL/DME (v/v = 1:1) and THE with different volume ratios of 20%, 40%, 45%, 50%, 55%, 60%, 65%, 70%, 75%, and 80% (THE, vt.%).

Characterization. The morphology of Li anodes and Al current collectors after cycling in various electrolytes was characterized with SEM measurements (Zeiss Supra, Germany). The elemental content was analyzed with mapping. The Li anodes after cycling in various electrolytes were studied by XPS (Thermo-Fisher, ESCALAB 250Xi, U.S.A.). Battery disassembling and electrode cutting were carried out and then sealed in the glove box. Then, the sealed sample was transferred into the XPS chamber, taken out rapidly in the chamber and vacuumed immediately to avoid

exposure and oxidation in air. FTIR instrument (Nicolet, is50, U.S.A.) was conducted to study the Li anodes surface properties. Electrolyte viscosity was measured using a rheometer (Waters, VIS403039900, U.S.A.). The contact angle of the electrolytes on PP separator and various electrodes was tested by an automatic contact angle measuring instrument (ZhongChen, JC2000D3M, China). The thermal stability of various electrolytes was tested by DSC (NETZSCH, STA 449F3, Germany). OCV curves were tested with a heating rate of 5 °C/min. Electrolyte uptake was tested with the PP separator. Contact angle measurement for different electrolytes was based on the PP separator. Ignition tests for different electrolytes were based on glass-fiber films.

The Li^+ transference number was defined as the equation (Equation (1)).

$$t_{Li^+} = \frac{\mu_{Li^+}}{\mu_{Li^+} + \mu_{TFSI^-}} \quad (1)$$

where μ_{Li^+} is the mobility of Li^+ and μ_{TFSI^-} is the mobility of $TFSI^-$.

The conductivity inside electrolyte was defined as the equation (Equation (2)).

$$\sigma_{electrolyte} = n_{Li^+} e \mu_{Li^+} + n_{TFSI^-} e \mu_{TFSI^-} \quad (2)$$

where n_{Li^+} and n_{TFSI^-} are the numbers of anion and cation per unit volume, respectively, μ_{Li^+} and μ_{TFSI^-} are the mobilities of anion and cation, respectively, and e is electronic charge.

The conductivity at electrolyte/anode interface in the 60%THE electrolyte was defined as the equation (Equation (3)).

$$\sigma_2 = (1-\alpha)\sigma_1 + \alpha\sigma_{LiF} \quad (3)$$

where σ_1 is the ion conductivity at electrolyte/anode interface in commercial electrolyte, σ_{LiF} is the ion conductivity of LiF and α denotes as the increased ratio of F element after introducing THE.

The Li^+ transference numbers of various electrolytes were studied with AC impedance and DC polarization analysis. The polarization currents, referred to the initial current (I_0) and steady-state current (I_S) of the Li/Li cell, were obtained with a polarization potential (ΔV) at 10 mV. In addition, the initial and steady-state interfacial resistances (R_O and R_S) of Li/electrolyte were determined through the impedance measurements before and after the potentiostatic polarization. The impedance measurements were performed at an open-circuit potential in a frequency range from 0.10 Hz to 1.0 MHz. $t_{Li}{}^+$ was calculated according to the Bruce-Vincent-Evans equation[1] (Equation (4)):

$$t_{Li^+} = \frac{I_S(\Delta V - I_O R_O)}{I_O(\Delta V - I_S R_S)} \quad (4)$$

The equation[1] (Equation (5)) of the conductivity inside electrolyte was given:

$$\sigma_{electrolyte} = \frac{L}{SR_e} \quad (5)$$

where L is the thickness of the separator and S is the area of the electrode. *Re* is the Ohmic resistance, which was tested at an open-circuit potential in a frequency range from 0.10 Hz to 1.0 MHz.

Electrochemical measurements. For the fabrication of electrodes, active substance (LFP, LCO, NCM523, NCM811, and LTO), conductive carbon, and binder (PVDF in

N-methyl pyrrolidone (NMP)) were mixed with a weight ratio of 8:1:1. 90 wt.% graphite, 5 wt.% conductive carbon and 5 wt.% binder (2.5 wt.% sodium carboxymethyl cellulose and 2.5 wt.% styrene-butadiene rubber) constituted graphite anode. The cathode slurry was coated on aluminum foil and dried at 60 ºC for 12 h. The anode slurry was coated on copper foil and dried at 80 ºC for 12 h. The resultant electrodes were cut into a circular sheet of 14 mm in diameter (In full cells, LCO, LFP, NCM523 and NCM811 electrodes were cut into a circular sheet of 12 mm in diameter). The loading mass of the regular LFP electrodes was 0.72-1.08 mg cm^{-2}. The loading mass of the high-loading LFP electrodes was 13.00-13.67 mg cm^{-2}. The loading mass of the LTO electrodes was 10.16-11.52 mg cm^{-2}. The loading mass of the LCO electrodes was 11.17-11.35 mg cm^{-2}. The loading mass of the NCM523 electrodes was 14.71-14.86 mg cm^{-2}. The loading mass of the NCM811 electrodes was 12.71-12.79 mg cm^{-2}. The loading mass of the graphite electrodes was 4.28-7.51 mg cm^{-2}. For all full cells, the capacity ratio of the negative to the positive electrodes (N/P ratio) was set to 1.05. The LIBs were tested in 2035 coin-type cells. The amount of electrolyte used was 45 μL (For the high-loading electrodes, the amount of electrolyte used was 75 μL). The separator was Celgard 2500 with the diameter of 16 mm. Galvanostatic measurements were performed on the battery testing system (Neware, CT-3008), within the voltage range of 2.5-4.2 V (*vs.* Li^+/Li) for Li/LFP cells, within the voltage range of 1.0-2.5 V (*vs.* Li^+/Li) for Li/LTO cells, within the voltage range of 2.5-3.8 V (*vs.* Li^+/Li) for graphite/LFP cells, within the voltage range of 0.5-2.8 V (*vs.* Li^+/Li) for LTO/LFP cells, within the voltage range of 3.0-4.4 V (*vs.*

Li^+/Li) for graphite/LCO cells, within the voltage range of 3.0-4.6 V (*vs.* Li^+/Li) for graphite/NCM523 cells, and within the voltage range of 2.5-4.3 V (*vs.* Li^+/Li) for graphite/NCM811 cells. Nyquist plots for the impedances of various electrolytes at different temperatures were tested in a frequency range from 0.10 Hz to 1.0 MHz (Shanghai Chenhua, CHI 760e, China). The CV analysis of the Li/LFP batteries was conducted in a voltage range of 2.5−4.2 V at a scan rate of 0.1 mV s^{-1}.

Electrolyte uptake test: The electrolyte uptake was calculated through the following equation (6).

$$\text{Electrolyte uptake (\%)} = \frac{m_{wet}-m_{dry}}{m_{dry} * \rho_{electrolyte}} \times 100 \tag{6}$$

where m_{wet} and m_{dry} denote the weight of wet and dry separator, respectively. $\rho_{electrolyte}$ represents the electrolyte density. To eliminate the influence of different electrolyte densities, the mass of dry separator was multiplied with the electrolyte density to obtain the corresponding reference mass. In every 5 minutes, the mass of the wet separator with adsorbed electrolyte was weighed and divided by the reference mass to obtain the percentage of electrolyte uptake. The density calculation of various electrolytes was as follows:

$\rho_{60\%THE\ electrolyte} = m_{60\%THE\ electrolyte} / V_{60\%THE\ electrolyte} = m_{LiTFSI} + m_{THE} + m_{DOL} + m_{DME} / V_{60\%THE\ electrolyte} = m_{LiTFSI} + \rho_{THE}V_{THE} + \rho_{DOL}V_{DOL} + \rho_{DME}V_{DME} / V_{60\%THE\ electrolyte} = 0.2871 + 1.5398 \times 0.6 + 1.0655 \times 0.2 + 0.8665 \times 0.2 / 1 = 1.5974\ g/cm^3$

$\rho_{DOL+DME\ electrolyte} = m_{DOL+DME\ electrolyte} / V_{DOL+DME\ electrolyte} = m_{LiTFSI} + m_{DOL} + m_{DME} / V_{DOL+DME\ electrolyte} = m_{LiTFSI} + \rho_{DOL}V_{DOL} + \rho_{DME}V_{DME} / V_{DOL+DME\ electrolyte} = 0.2871 + 1.0655 \times 0.5 + 0.8665 \times 0.5 / 1 = 1.2531\ g/cm^3$

$\rho_{EC+DMC\ electrolyte} = m_{EC+DMC\ electrolyte} / V_{EC+DMC\ electrolyte} = m_{LiPF6} + m_{EC} + m_{DMC} / V_{EC+DMC\ electrolyte} = m_{LiPF6} + \rho_{EC}V_{EC} + \rho_{DMC}V_{DMC} / V_{EC+DMC\ electrolyte} = 0.1519 + 1.3218\times0.5 + 1.0690\times0.5 / 1 = 1.3473$ g/cm^3.

***In-situ* Raman Spectroscopy.** *In-situ* Raman measurements were taken through Renishaw InVia Raman microscopy with a 532 nm laser irradiating the electrolyte through a modified negative case and Li metal foil with a 5 mm hole. The batteries were assembled in an argon-filled DELLIX glove box with oxygen and water levels both below 0.1 ppm. After aging treatment, galvanostatic measurements were performed at 0.2 C within the voltage range of 2.5-4.2 V for Li/LFP cells while the Raman signals were recorded simultaneously. The peaks of the spectra were normalized by subtracting the baseline.

DFT calculations. The HOMO and LUMO values of DOL, DME and THE molecules were calculated with Dmol^3 package. The absorption energy of electrolyte and separator was calculated using DFT implemented with DMol^3 package. To prove the better wettability and safety, the binding configurations of THE-$CH_3CH_2CH_3$, DOL-$CH_3CH_2CH_3$, DME-$CH_3CH_2CH_3$, THE-oxygen radical, DOL-oxygen radical, DME-oxygen radical, EC-oxygen radical, and DMC-oxygen radical were built and calculated. The adsorption energy was calculated with the equation: $E = E_{a\text{-}b} - (E_a + E_b)$, where E is the adsorption energy, and $E_{a\text{-}b}$ is the total energy of the relaxed a and b models at the equilibrium state. E_a and E_b are the self-consistent field (SCF) calculation energy values of geometry-optimized a and b models. Electron exchange correlation was constructed by Perdew-Burke-Ernzerhof (PBE) function

with generalized gradient approximation (GGA). The calculation of binding energy between Al^{3+} and different solvents adopted the same calculation methods and conditions as described above.

The breaking energy of C-F bond was calculated through a $DMol^3$ package with the equation: $E_{C\text{-}F} = (E_{electrolyte(\text{-}F)} + E_F)\text{-}E_{electrolyte(e)}$, where $E_{C\text{-}F}$ is the breaking energy of C-F bond, and $E_{electrolyte(e)}$ is the electrolyte molecule system with a single electron. $E_{electrolyte(\text{-}F)}$ and E_F are the energy values of geometry-optimized electrolyte molecule with C-F bond cleavage and F anion, respectively.

AIMD calculations. Three electrolyte cells- a single $LiPF_6$ molecule dissolved in 7 EC and 7 DMC molecules, a single LiTFSI molecule dissolved in 5 DME and 7 DOL molecules, and a single LiTFSI molecule dissolved in 2 DME, 3 DOL and 4 THE molecules- were constructed in periodic boxes. We performed the AIMD calculation using the VASP package to understand Li^+ diffusion behavior. The ion–electron interaction was described with the projector augmented wave method, and the exchange-correlation energy was described by the functional of the Perdew–Burke–Ernzerhof form of the generalized gradient approximation. The plane wave energy cut-off of 400 eV was chosen and a minimal Γ-centred 1 × 1 × 1 k-point grid was used. All molecular dynamics simulations were performed in the NVT ensemble using a Nosé−Hoover thermostat. Each system was heated to 300 K and equilibrated for 10 ps and then simulated for 30 ps to obtain statistics.

Constrained AIMD simulations were also performed on the electrolyte/electrode

systems to understand rate performance. The deposited Li (0 0 1)/electrolyte interfaces were modelled by packing the DOL, DME and THE in the deposited Li (0 0 1) box, respectively. In these simulations, a slow-growth method was used, in which a Li^+ within the electrolyte was chosen and constrained to a position from the deposited Li (0 0 1) surface. The force required to constrain the Li^+ at this particular position was monitored. The shifted distance was 9.92×10^{-4} Å in every simulation step for approaching from the electrolyte to the surface, and the free energy of the system can be obtained by integrating the position dependent mean constraint force.

MD calculations. MD simulations were conducted on the electrolytes using the LAMMPS simulation package. OPLS-AA (optimized potentials for liquid simulations)[2,3] parameters and charges were generated and calculated by the LigParGen[4,5] for the solvent molecules. The parameters for $TFSI^-$ were taken from previous publications.[6] For LiTFSI in DOL/DME (v/v = 1:1) electrolyte, 30 LiTFSI, 150 DME, and 210 DOL were dissolved into a periodic box, while for LiTFSI in 60%THE electrolyte, 30 LiFSI, 60 DME, 90 DOL, and 120 THE molecules were calculated. The systems were set up initially with simulation boxes 50 Å in length, with the salt and solvent molecules distributed in the simulation boxes using Packmol.[7] First, NPT runs were performed at 300 K for 5.0 ns to ensure that the equilibrium salt dissociation had been reached. Then, the NPT runs were 3.0 ns long at 300 K and the last 2.0 ns were used to obtain the structure of the electrolyte.

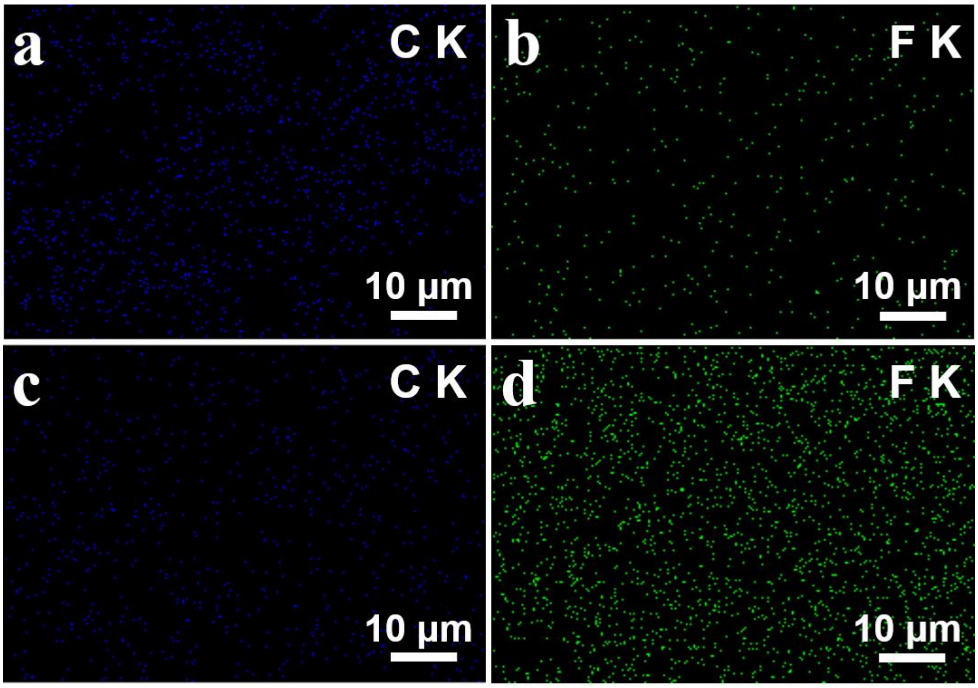

Supplementary Figure 1 | Energy-dispersive spectroscopy (EDS) test for the elemental mapping of (Figs. 1c and 1g) Li anodes in (a and b) DOL+DME and (c and d) 60%THE electrolytes after cycling. Elements of C and F are shown in the mapping.

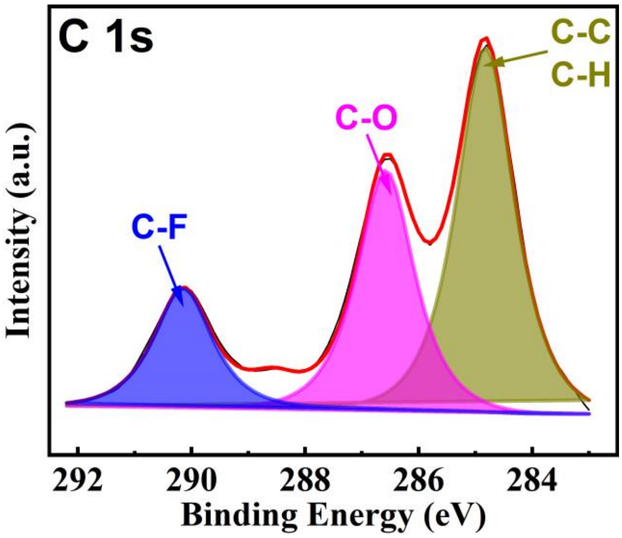

Supplementary Figure 2 | XPS of the SEI layer in the DOL+DME electrolyte, C 1s is presented, including peak deconvolution and assignments.

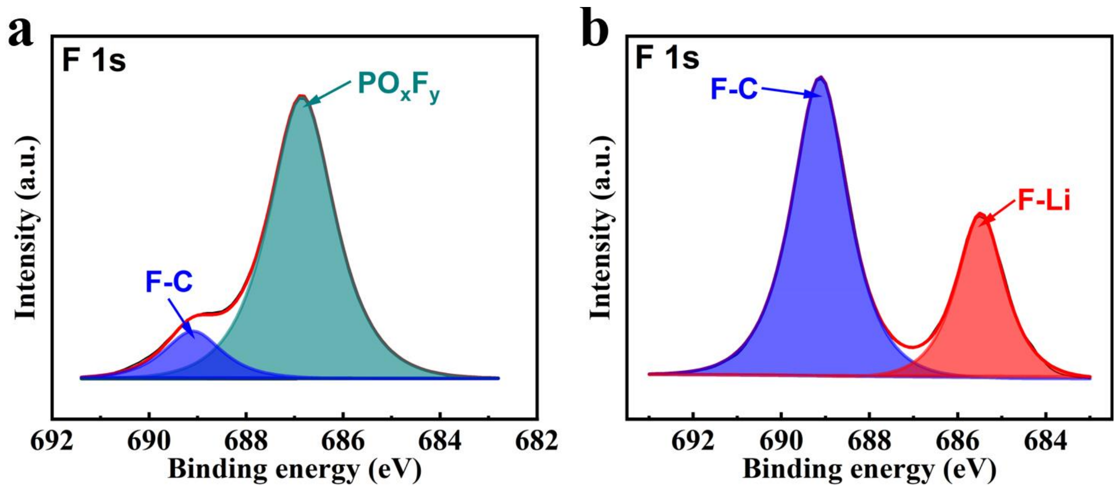

Supplementary Figure 3 | XPS of the SEI layer in (a) EC+DMC and (b) 60%THE electrolytes after 50 cycles at 60 °C, F 1s is presented, including peak deconvolution and assignments.

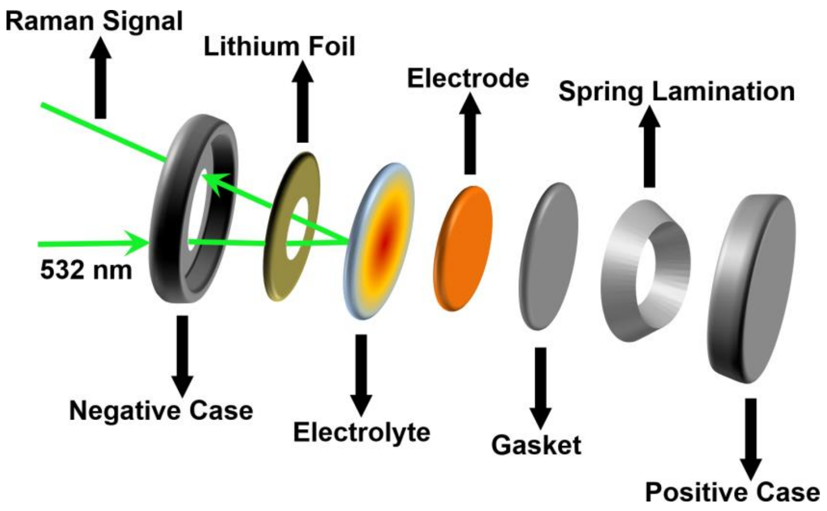

Supplementary Figure 4 | The cell configuration for *in-situ* Raman spectroscopic analysis.

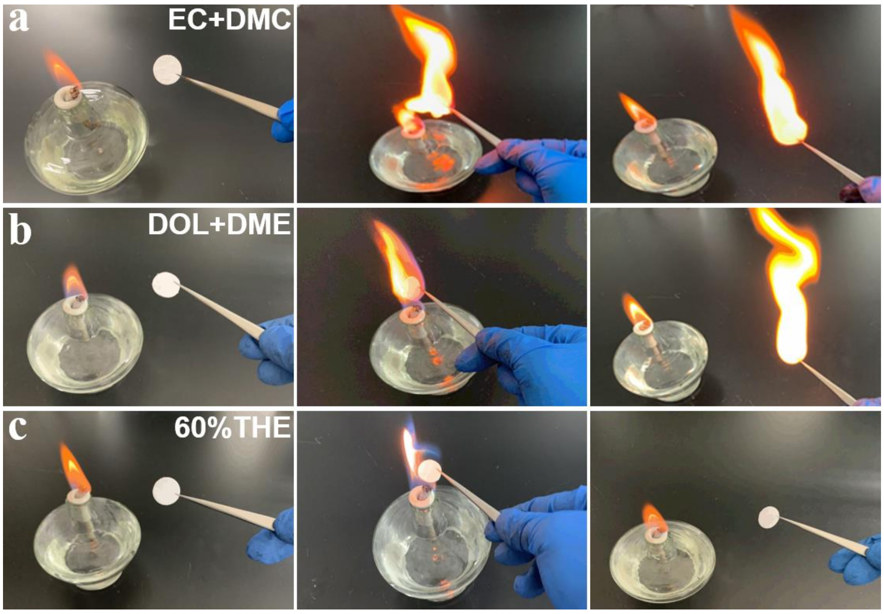

Supplementary Figure 5 | Ignition tests for (a) EC+DMC, (b) DOL+DME, and (c) 60%THE electrolytes on glass-fiber films.

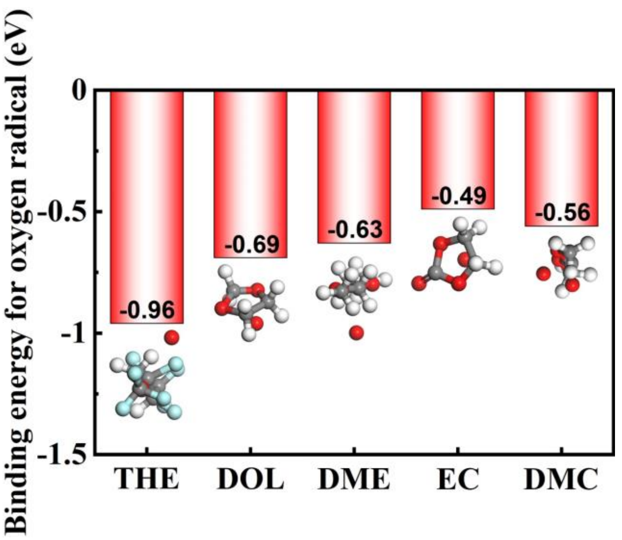

Supplementary Figure 6 | Binding energy between various solvents and oxygen radical.

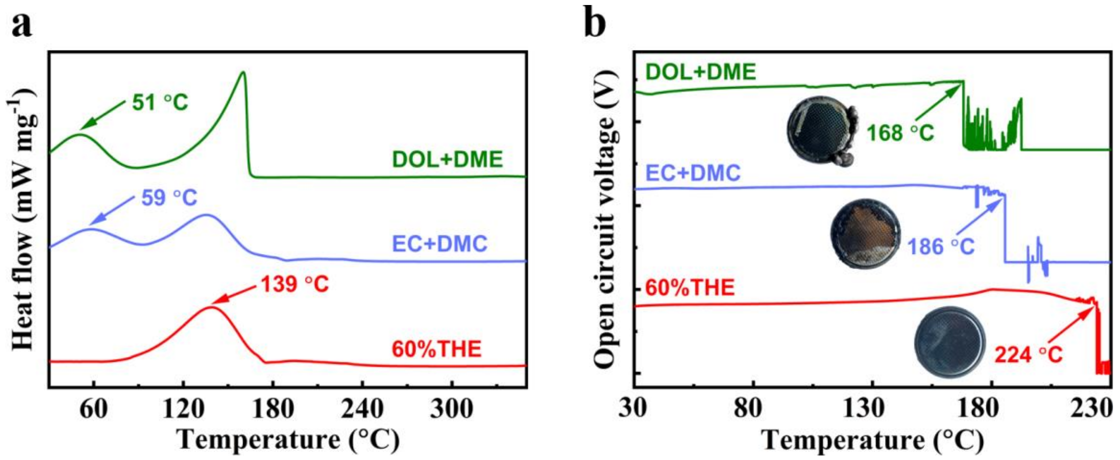

Supplementary Figure 7 | (a) DSC traces of DOL+DME, EC+DMC, and 60%THE electrolytes. (b) OCV curves of the Li/LFP cells with DOL+DME, EC+DMC, and 60%THE electrolytes with respect to temperature. Insets in the Figure show the optical images of the cells with different electrolytes after heating to 230 °C.

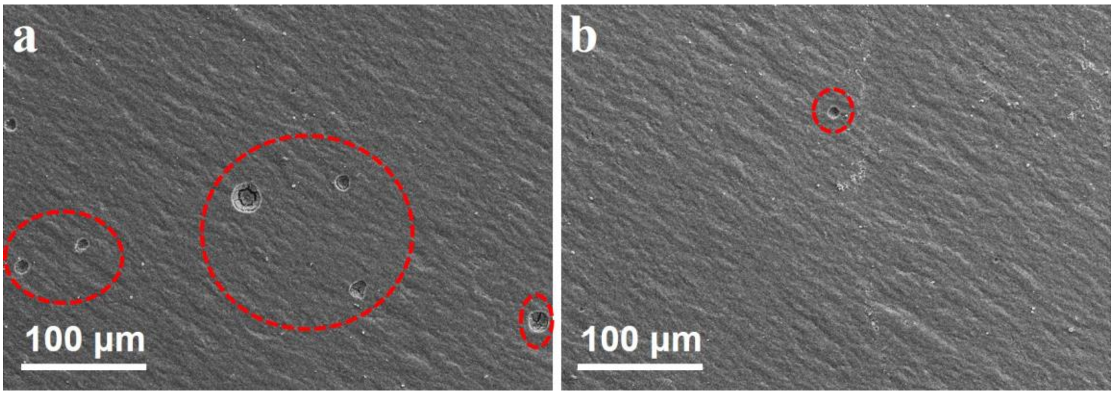

Supplementary Figure 8 | The SEM images of the aluminum current collector after being held at 4.5 V for 3 h in (a) DOL+DME and (b) 60%THE electrolytes.

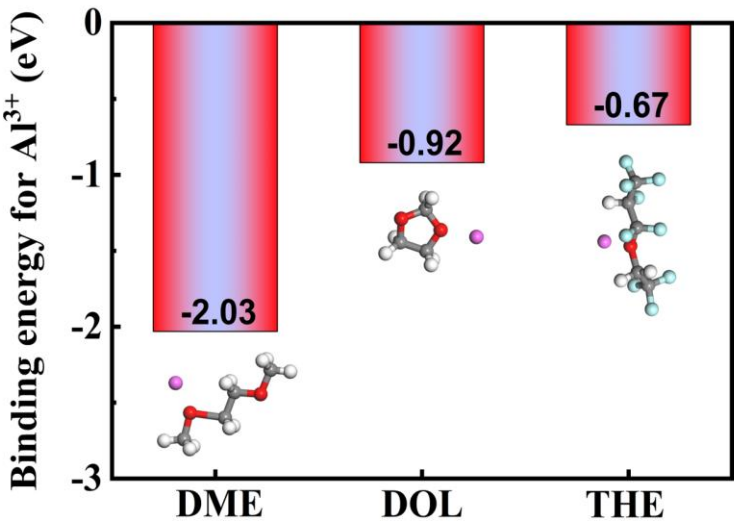

Supplementary Figure 9 | Binding energy between various solvents and Al^{3+}.

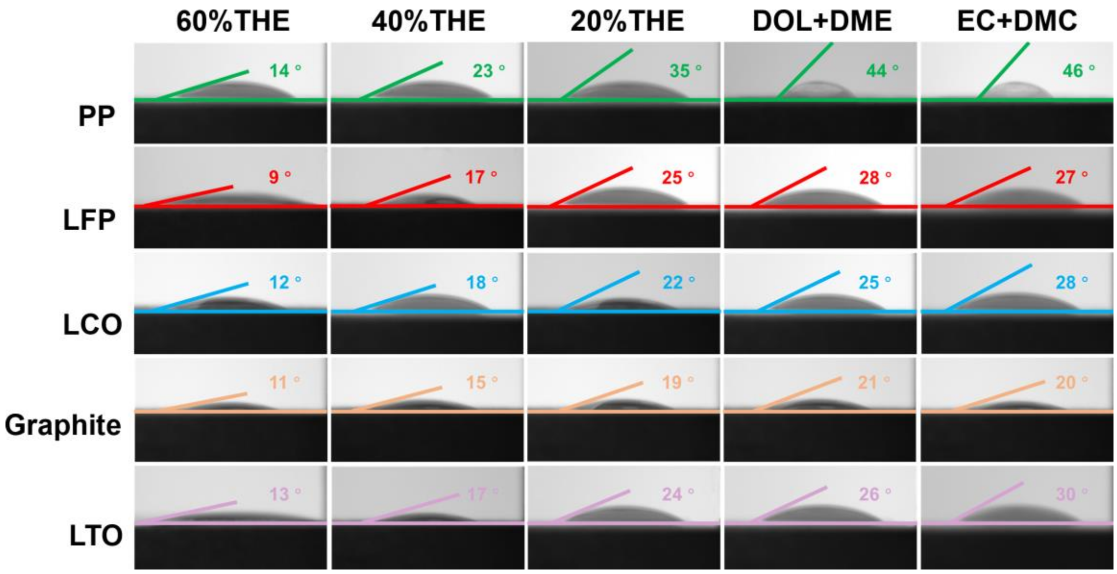

Supplementary Figure 10 | The digital photos of contact angles of different electrolytes to PP separator and various electrodes.

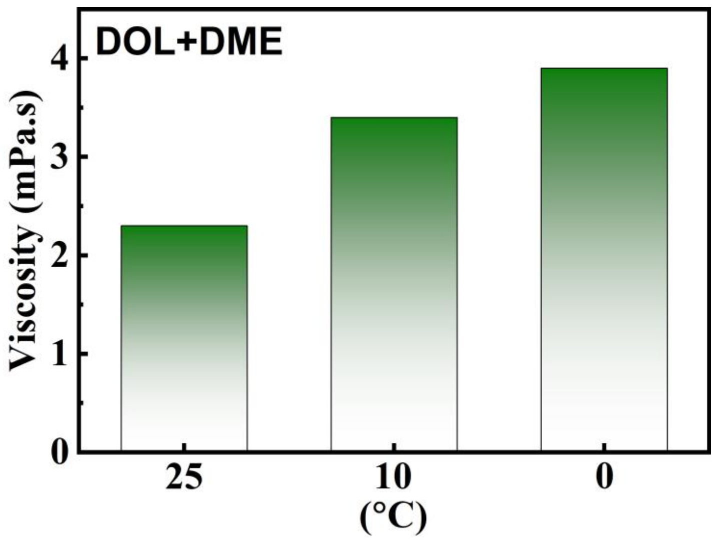

Supplementary Figure 11 | Viscosity of the DOL+DME electrolyte at different temperatures.

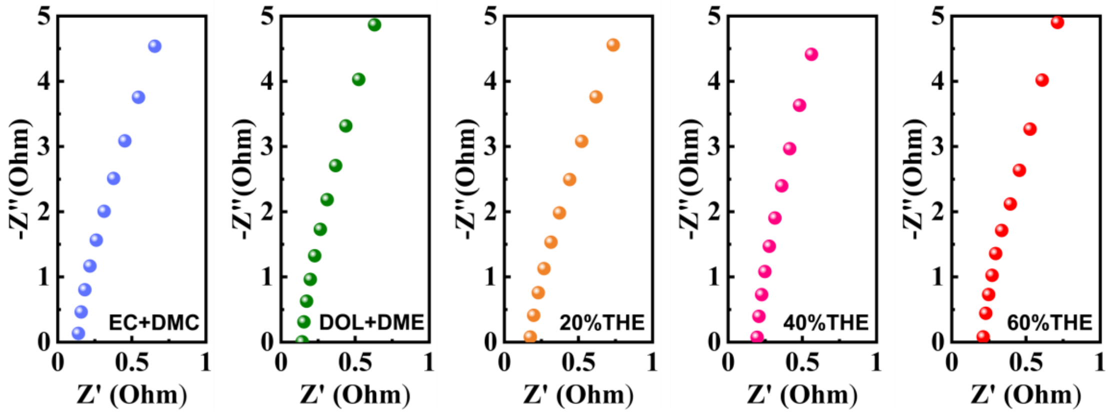

Supplementary Figure 12 | Nyquist plots for the impedances of EC+DMC, DOL+DME, 20%THE, 40%THE, and 60%THE electrolytes.

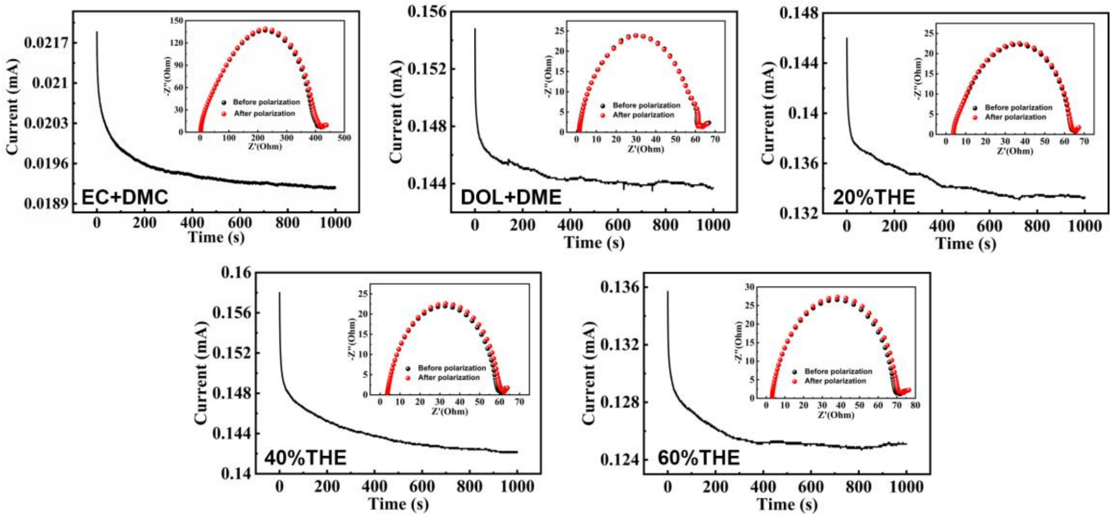

Supplementary Figure 13 | Current time profiles of the symmetrical Li/Li cells with EC+DMC, DOL+DME, 20%THE, 40%THE, and 60%THE electrolytes (the inset shows the Nyquist impedance spectra of the batteries before and after polarization).

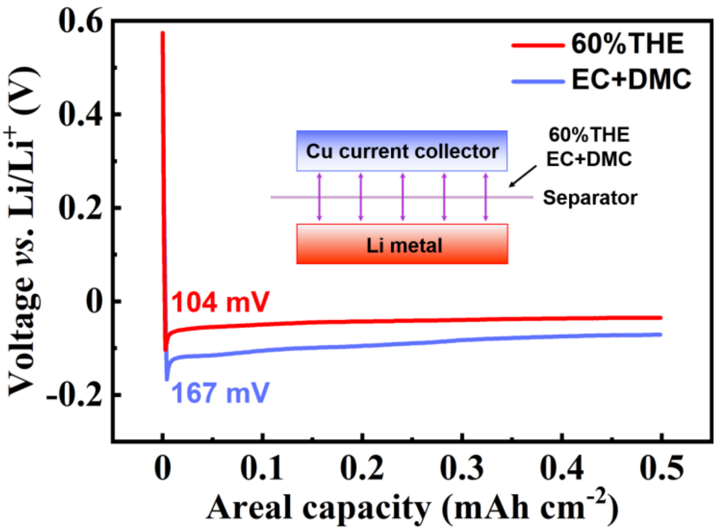

Supplementary Figure 14 | The nucleation overpotentials of 60%THE and EC+DMC electrolytes.

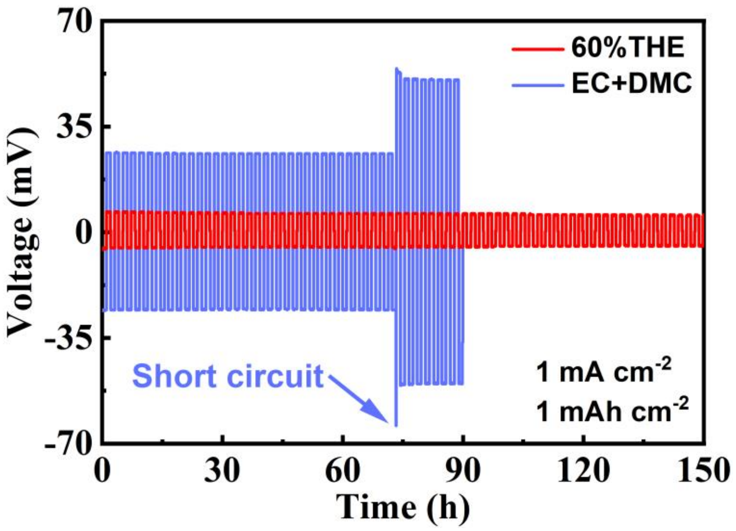

Supplementary Figure 15 | Cycling performances of the Li/Li cells using 60%THE and EC+DMC electrolytes at 1 mA cm^{-2} with a fixed capacity of 1 mAh cm^{-2}.

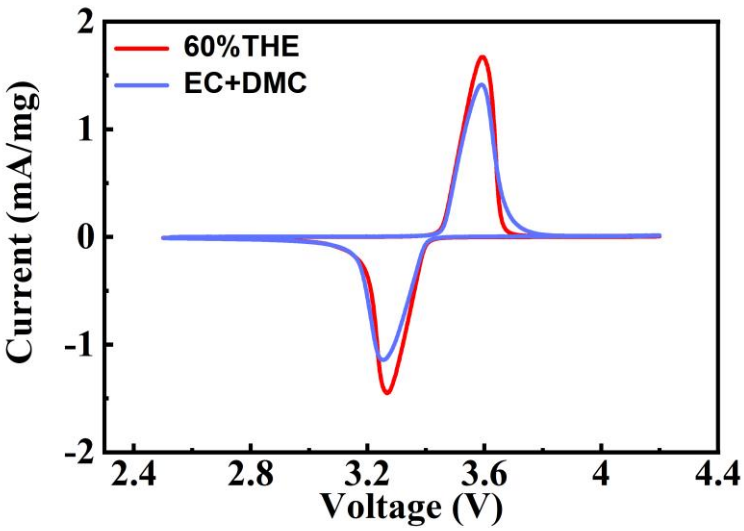

Supplementary Figure 16 | Cyclic voltammograms of the Li/LFP cells with EC+DMC and 60%THE electrolytes recorded at 0.1 mV s^{-1}.

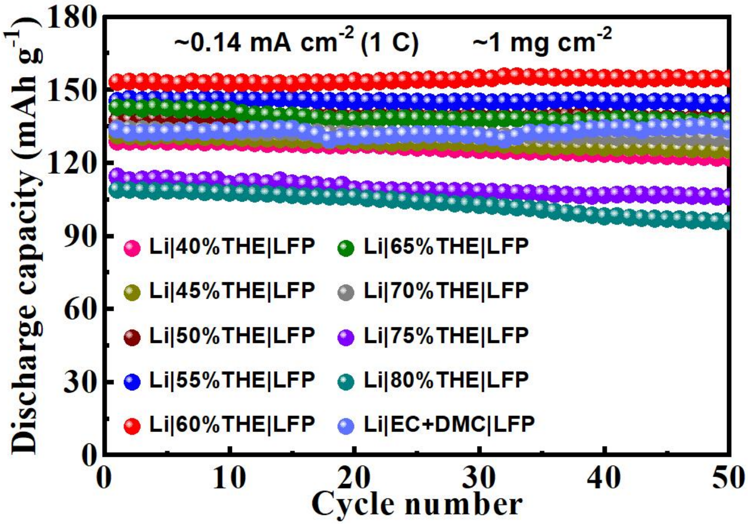

Supplementary Figure 17 | Cycling performances of the Li/LFP batteries with different electrolytes at 1 C. Ionic conductivity and Li^+ transference numbers are the key factors affecting the rate capacities. As shown in Fig. 2h, the 60%THE electrolyte exhibits not only a high Li^+ transference number of 0.62, but also an ionic conductivity comparable to commercial EC+DMC and DOL+DME electrolytes (that is, 6.2 mS cm^{-1} *vs.* 10.3 mS cm^{-1} and 9.2 mS cm^{-1}). The inferior ionic conductivity (2.9 mS cm^{-1} for the 80%THE electrolyte) limits the rate capacity, when the volume fraction of THE exceeds 60%; while the low Li^+ transference numbers (0.48 and 0.53 for 20%THE and 40%THE electrolytes, respectively) become the limiting factor, when the volume fraction of THE falls below 60%. Therefore, the 60%THE electrolyte leads to the highest capacity compared with other concentrations.

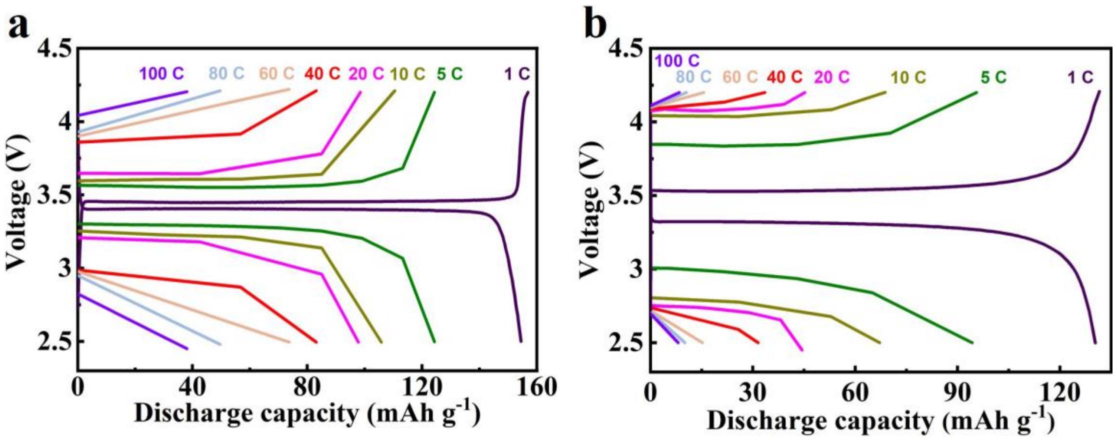

Supplementary Figure 18 | Voltage-capacity plots of the Li/LFP cells with (a) 60%THE and (b) EC+DMC electrolytes at different rates.

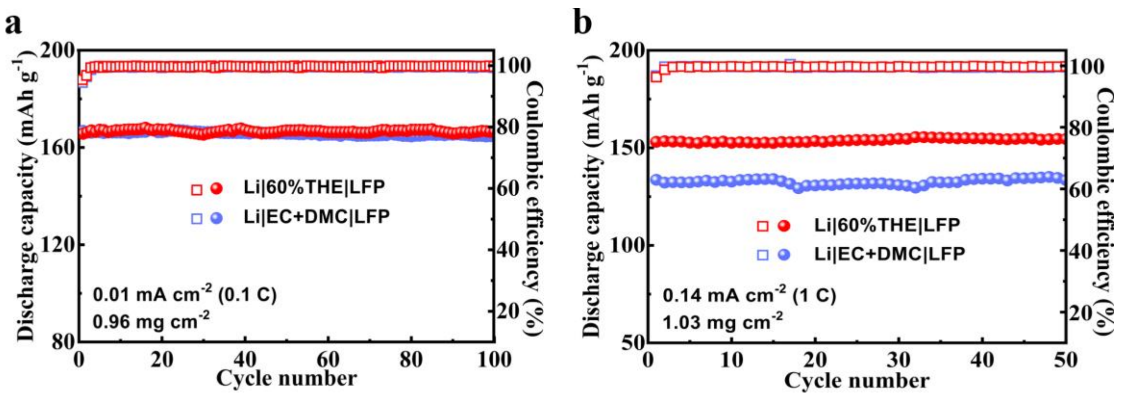

Supplementary Figure 19 | Cycling performances of the Li/LFP cells with EC+DMC and 60%THE electrolytes at (a) 0.1 C and (b) 1 C.

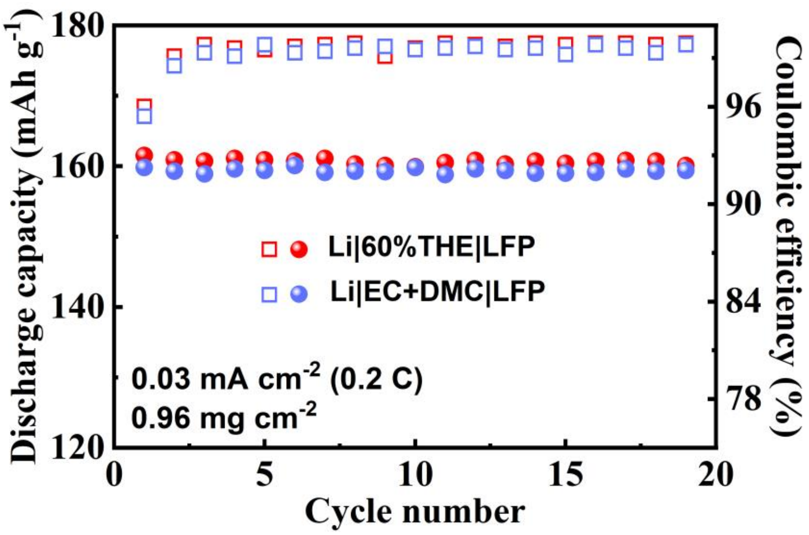

Supplementary Figure 20 | Cycling performances of the Li/LFP cells with EC+DMC and 60%THE electrolytes at 0.2 C.

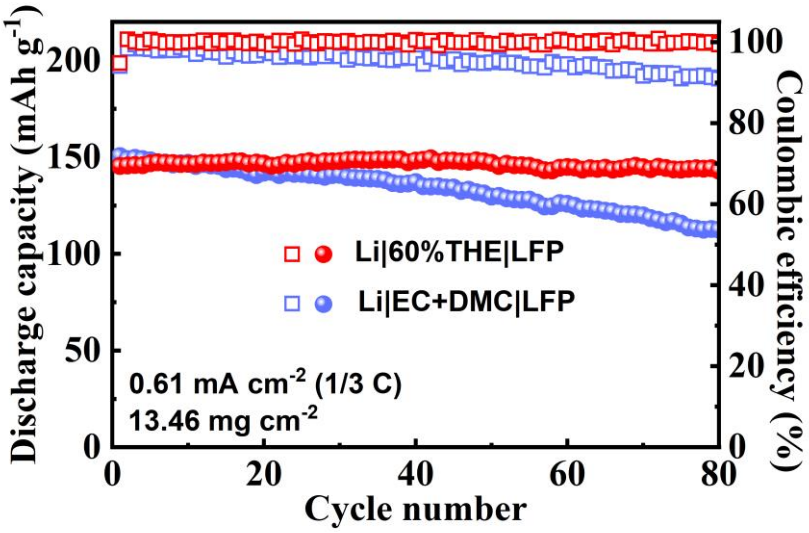

Supplementary Figure 21 | Cycling performances of the Li/LFP (1/3 C) batteries with EC+DMC and 60%THE electrolytes at high loading.

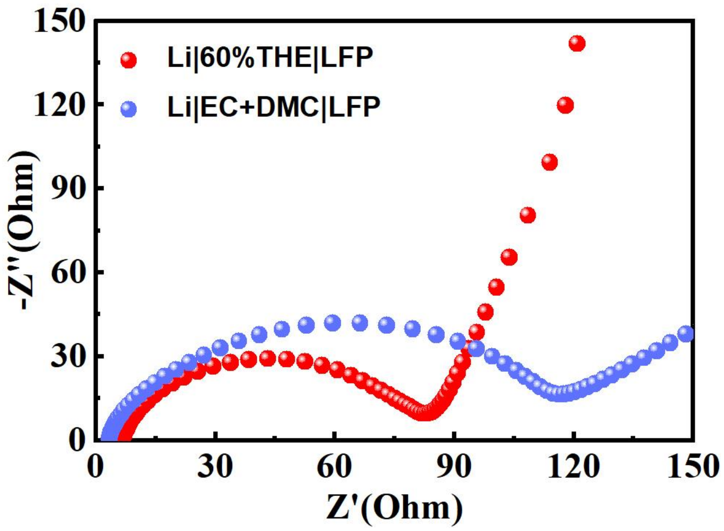

Supplementary Figure 22 | Nyquist impedance plots of the Li/LFP cells with EC+DMC and 60%THE electrolytes.

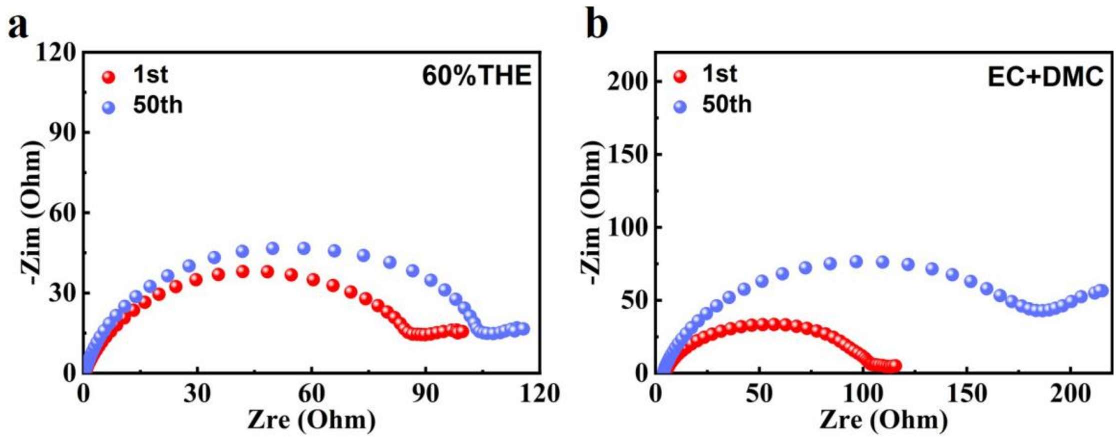

Supplementary Figure 23 | Nyquist plots of the Li/LFP cells with (a) 60%THE and (b) EC+DMC electrolytes after 1st and 50th cycles at 1 C.

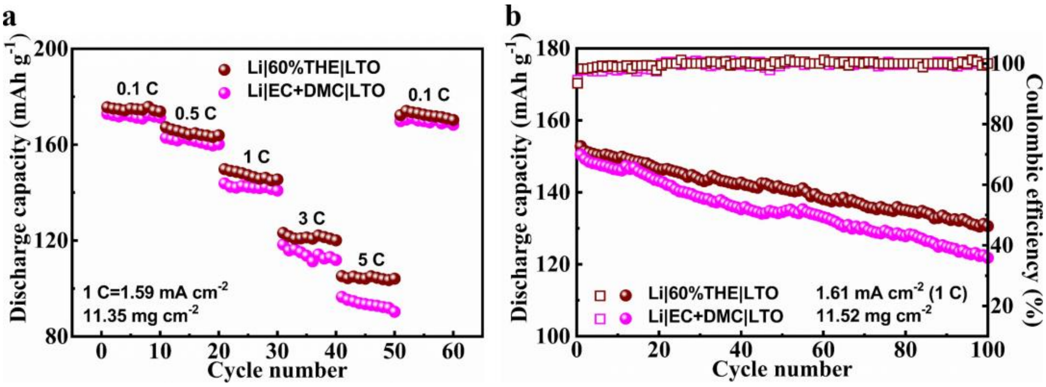

Supplementary Figure 24 | (a) Rate performance and (b) cycling performance (1 C) of the Li/LTO cells with EC+DMC and 60%THE electrolytes.

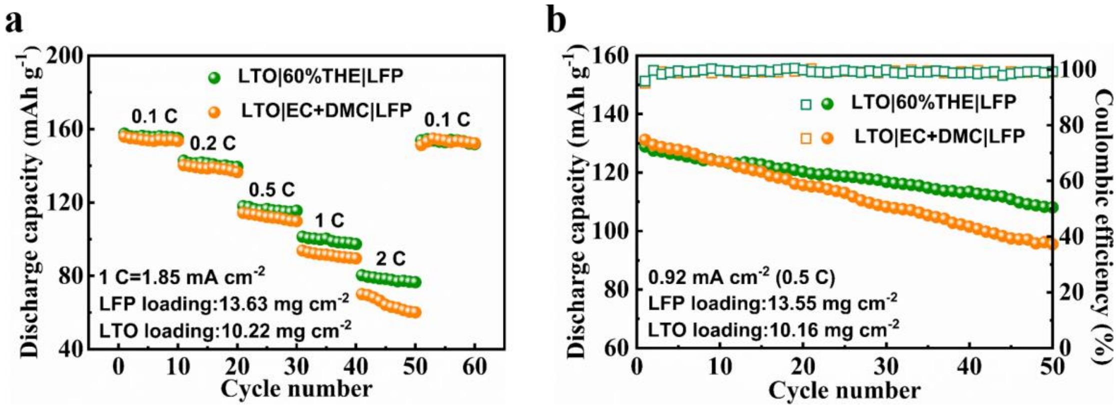

Supplementary Figure 25 | (a) Rate performance and (b) cycling performance (0.5 C) of the LTO/LFP cells with EC+DMC and 60%THE electrolytes.

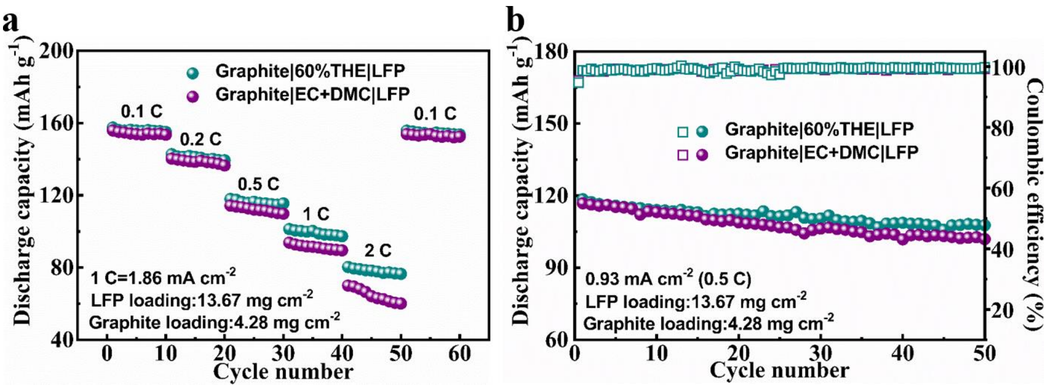

Supplementary Figure 26 | (a) Rate performance and (b) cycling performance (0.5 C) of the graphite/LFP cells with EC+DMC and 60%THE electrolytes.

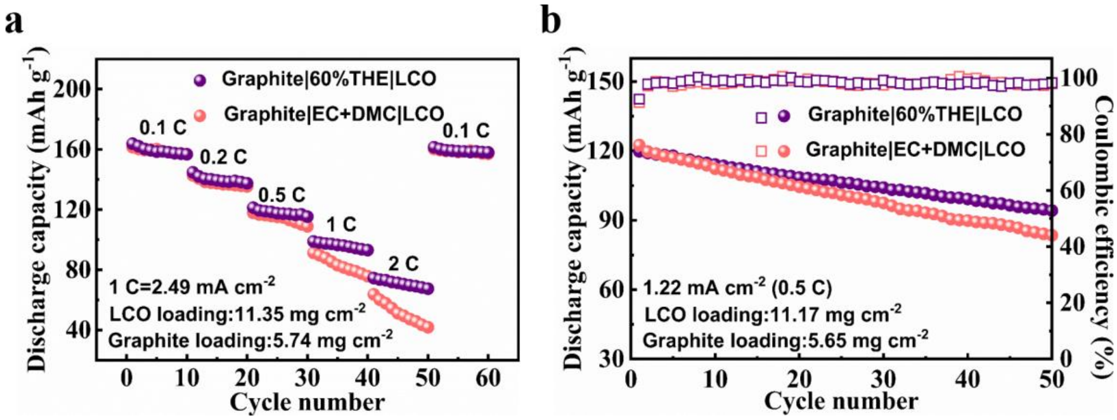

Supplementary Figure 27 | (a) Rate performance and (b) cycling performance (0.5 C) of the graphite/LCO cells with EC+DMC and 60%THE electrolytes.

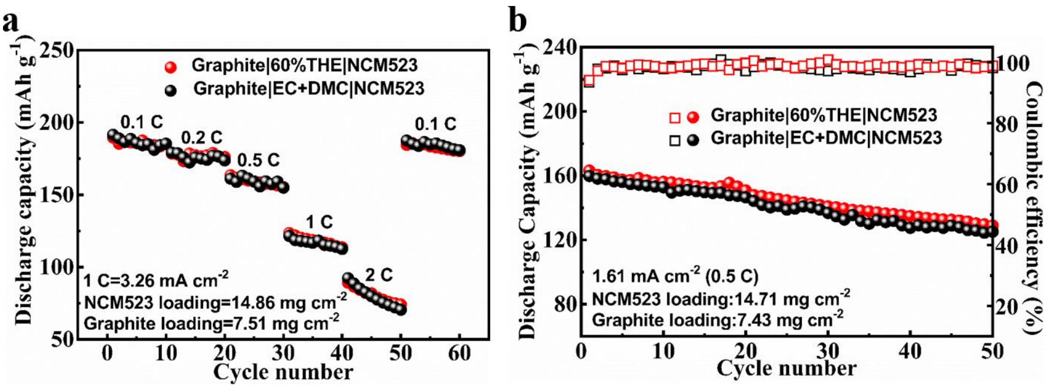

Supplementary Figure 28 | (a) Rate performance and (b) cycling performance (0.5 C) of the graphite/NCM523 cells with EC+DMC and 60%THE electrolytes.

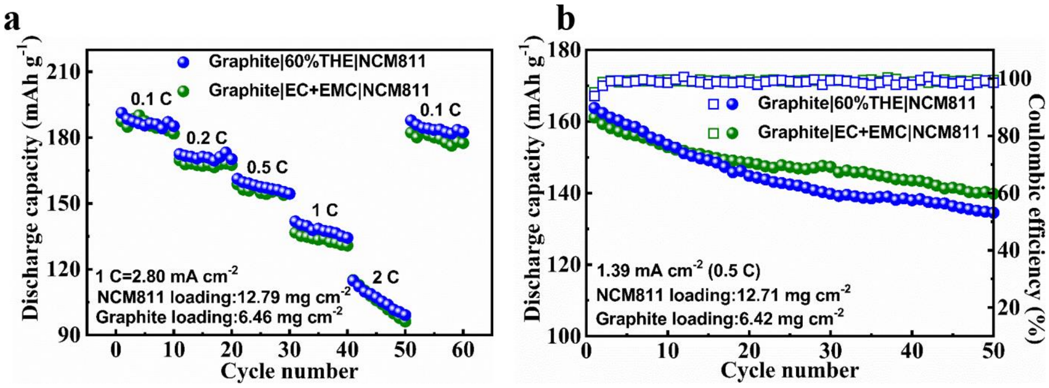

Supplementary Figure 29 | (a) Rate performance and (b) cycling performance (0.5 C) of the graphite/NCM811 cells with EC+EMC and 60%THE electrolytes.

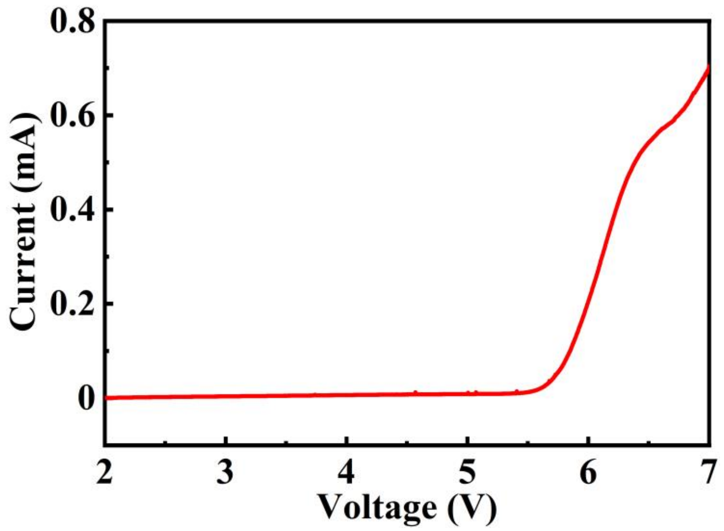

Supplementary Figure 30 | Electrochemical window of THE solvent.

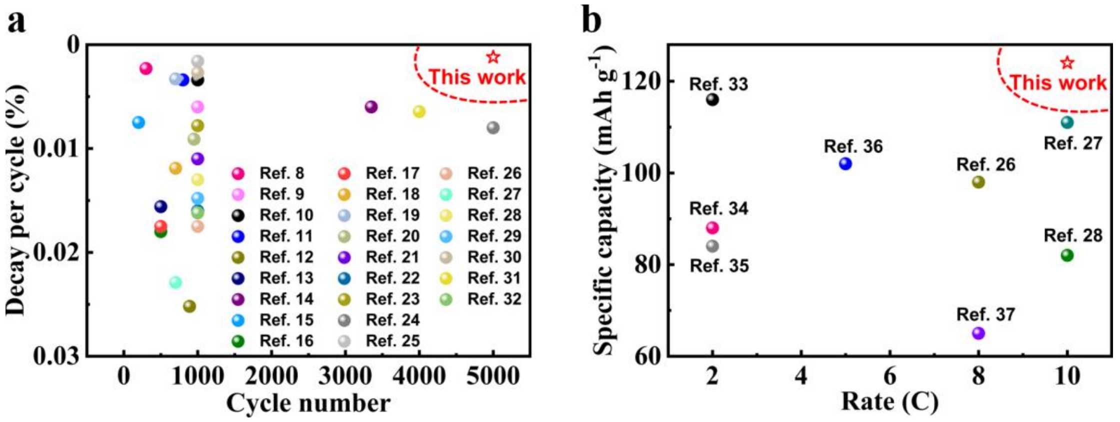

Supplementary Figure 31 | (a) Long-term cycling performance comparison of this work and recent relevant reports for Li/LFP cells. (b) Rate capacity comparison of this work and recent relevant reports on electrolyte engineering for Li/LFP cells.

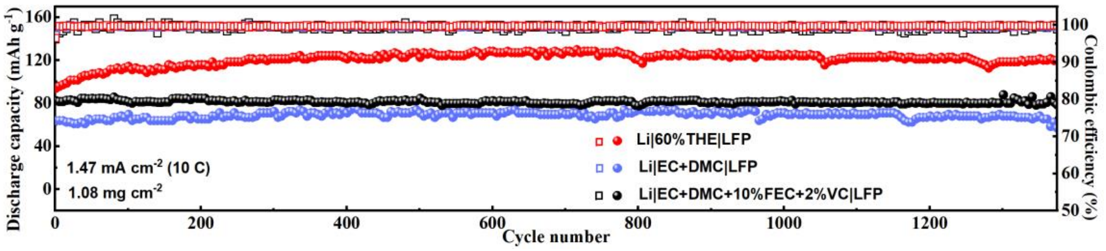

Supplementary Figure 32 | Long-term cycling performances of the Li/LFP cells with EC+DMC, EC+DMC+10%FEC+2%VC, and 60%THE electrolytes at 10 C.

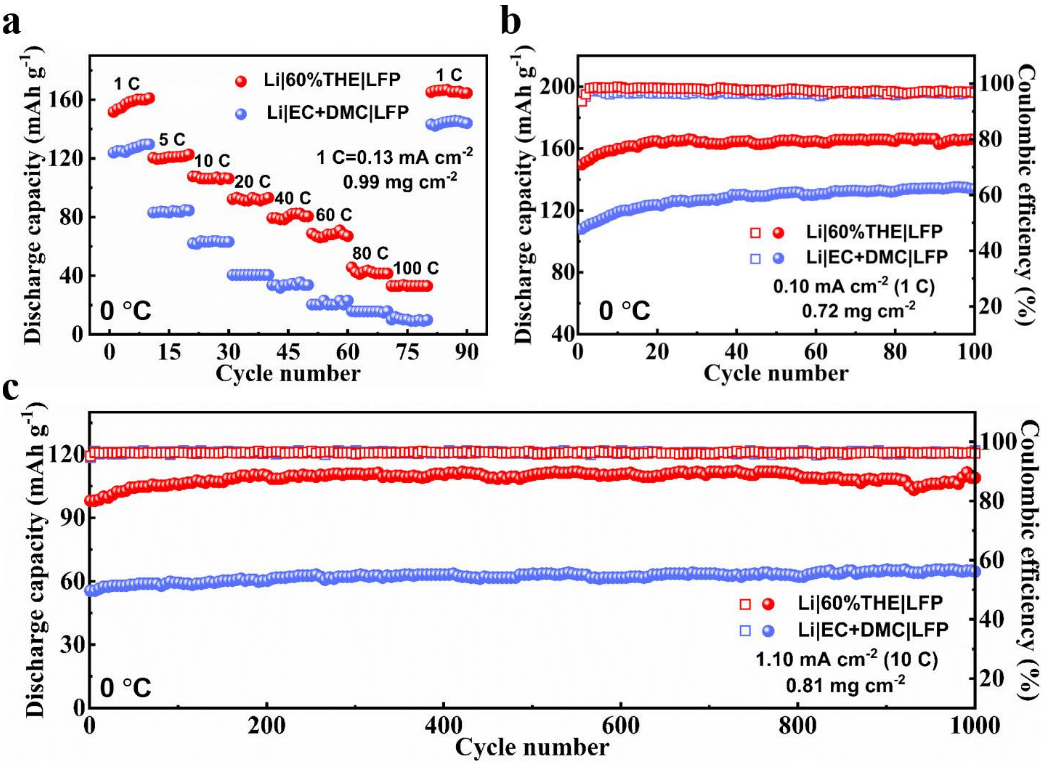

Supplementary Figure 33 | (a) Rate performance of the Li/LFP cells with EC+DMC and 60%THE electrolytes at low temperature (0 °C). Low temperature (0 °C) cycle performance of the Li/LFP cells with EC+DMC and 60%THE electrolytes at (b) 1 C and (c) 10 C.

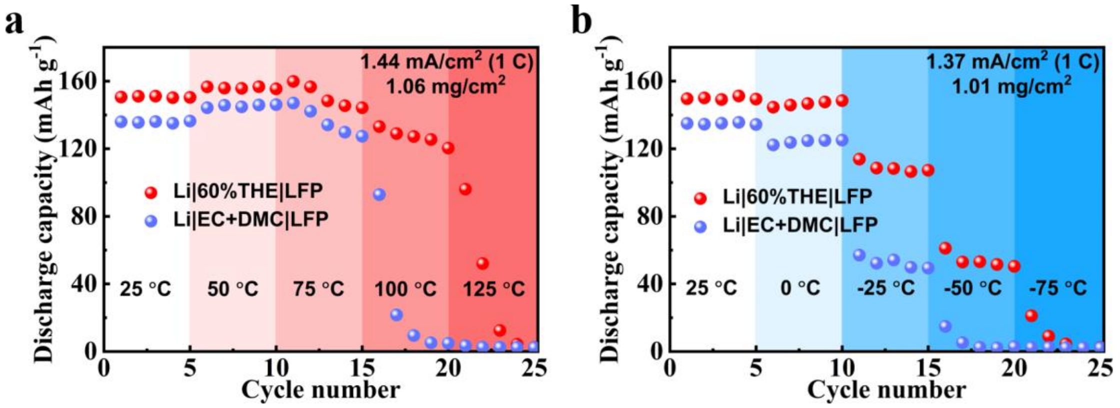

Supplementary Figure 34 | Cycling performance of the Li/LFP cells with EC+DMC and 60%THE electrolytes at different (a) high and (b) low temperatures.

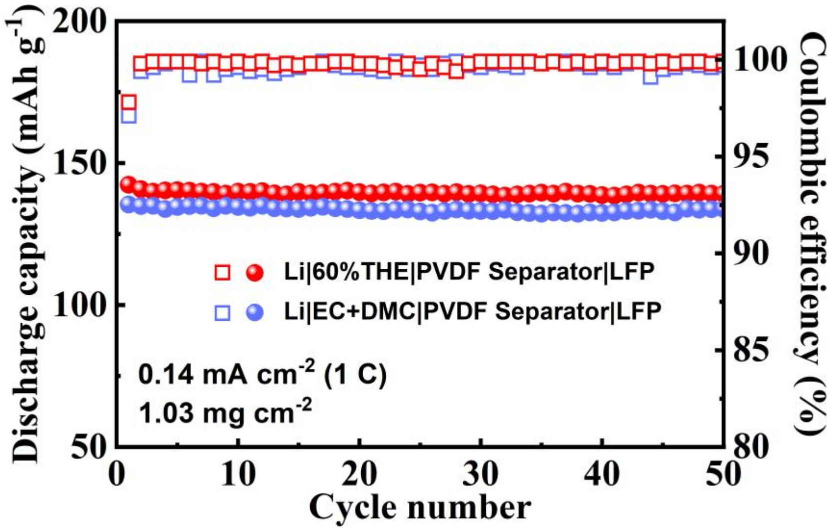

Supplementary Figure 35 | Cycling performances of the Li/LFP batteries with different electrolytes and PVDF separator at 1 C.

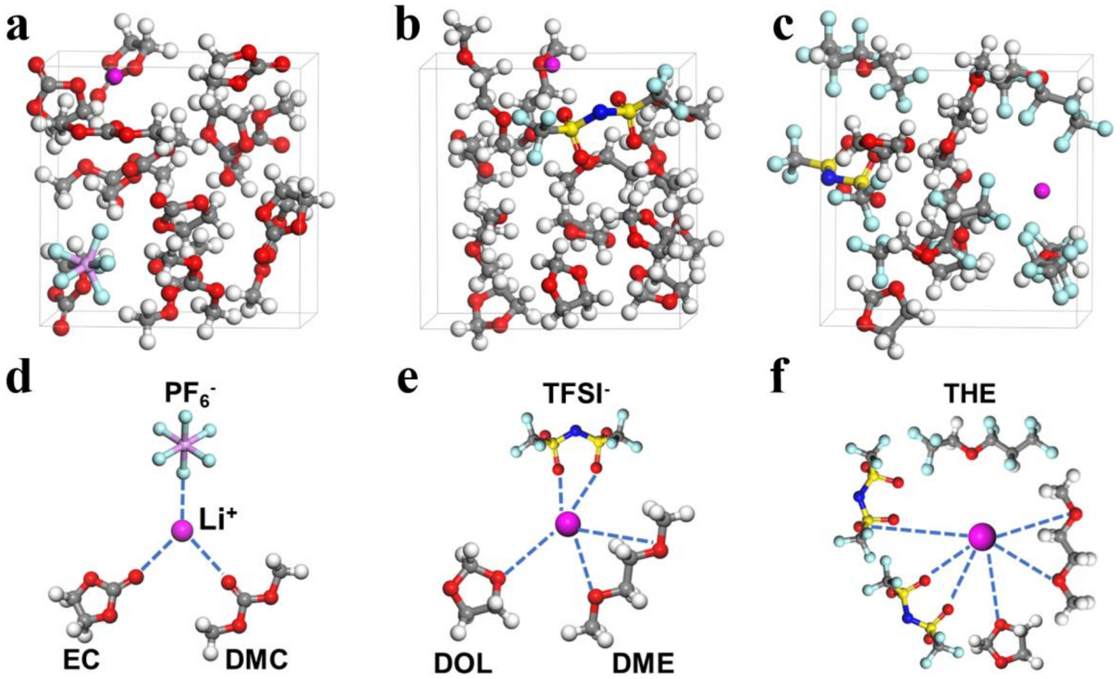

Supplementary Figure 36 | The representative AIMD simulation snapshots of (a) EC+DMC, (b) DOL+DME, and (c) 60%THE electrolytes respectively. The representative Li^+ solvation structures of (d) EC+DMC, (e) DOL+DME, and (f) 60%THE electrolytes respectively. Balls with various colors represent different atoms; color code: red, O; light green, P; blue, N; yellow, S; light blue, F; magenta, Li; grey, C; white, H.

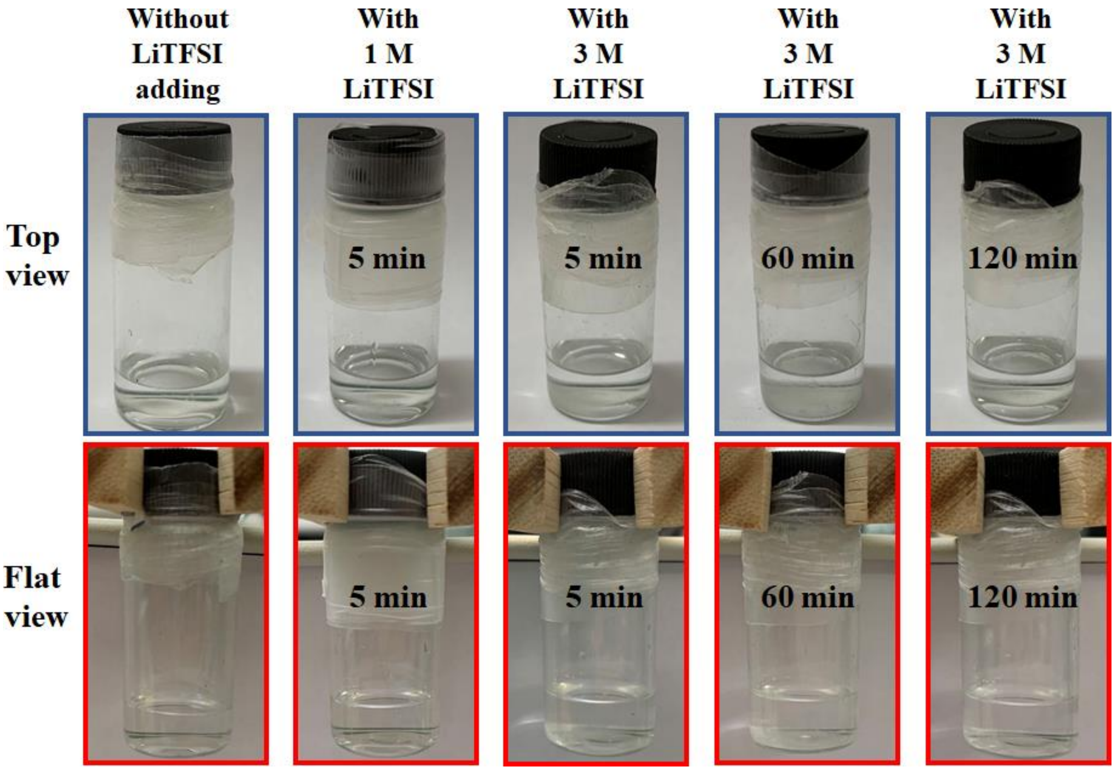

Supplementary Figure 37 | Digital photos of the 60 vt.% THE solvent without LiTFSI, with 1 M LiTFSI, and with 3 M LiTFSI. The 60 vt.% THE solvent without LiTFSI is not stratified, indicating that THE, DOL, and DME mix well. After 5 minutes, 1 M LiTFSI has been completely dissolved in the 60 vt.% THE solvent. To study the possibility of high salt concentration in the THE solvent, we further increase the LiTFSI concentration to 3 M. After 60 minutes, there is still a significant salt precipitation layer at the bottom of the electrolyte. After 120 minutes, 3 M LiTFSI is completely dissolved. Therefore, although THE does not coordinate with either Li^+ or anions, the 60%THE electrolyte still exhibits good lithium salt dissolving capability.

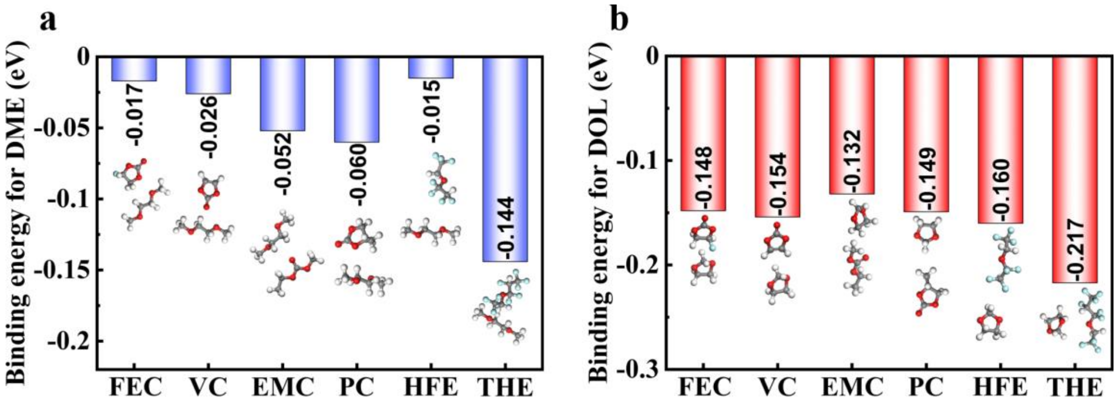

Supplementary Figure 38 | (a) Binding energy between various additive solvents and DME. (b) Binding energy between various additive solvents and DOL.

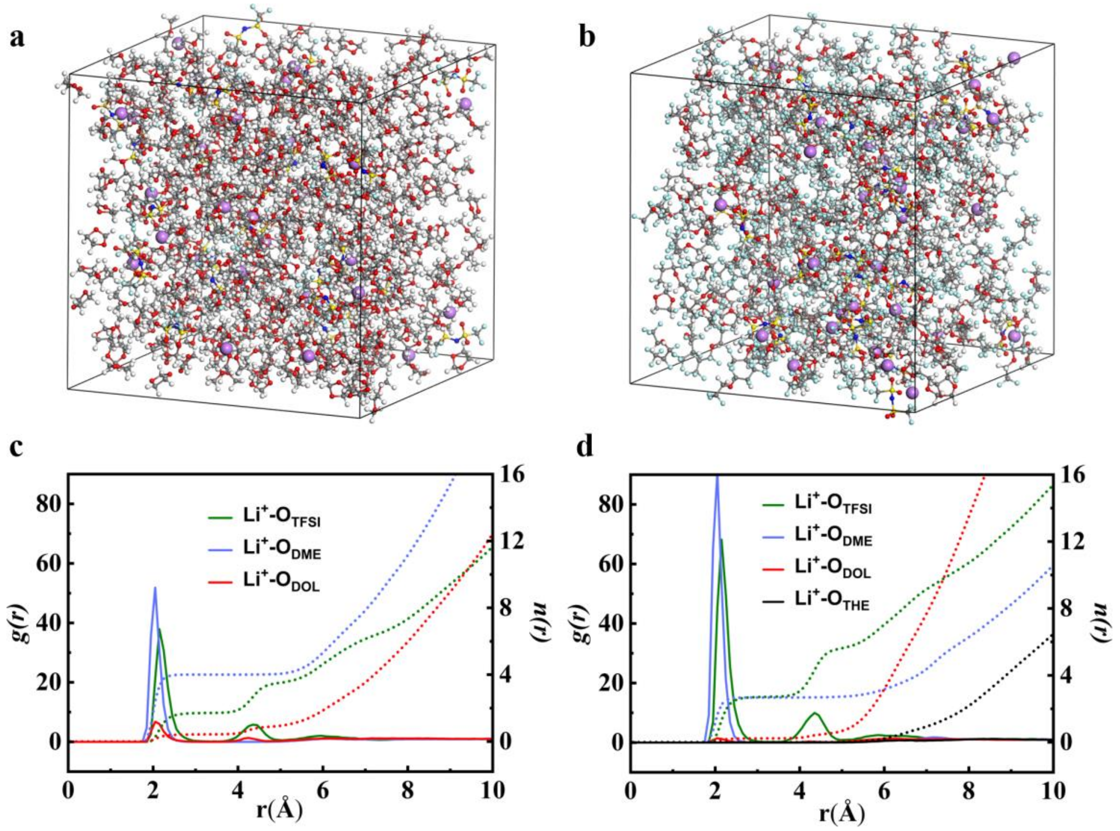

Supplementary Figure 39 | Snapshot obtained from MD simulations of DOL+DME (a) and 60%THE (b) electrolytes. Li^+ radial distribution function (*g(r)*, solid lines), and coordination numbers (*n(r)*, dashed lines) obtained from MD simulations of DOL+DME (c) and 60%THE (d) electrolytes.

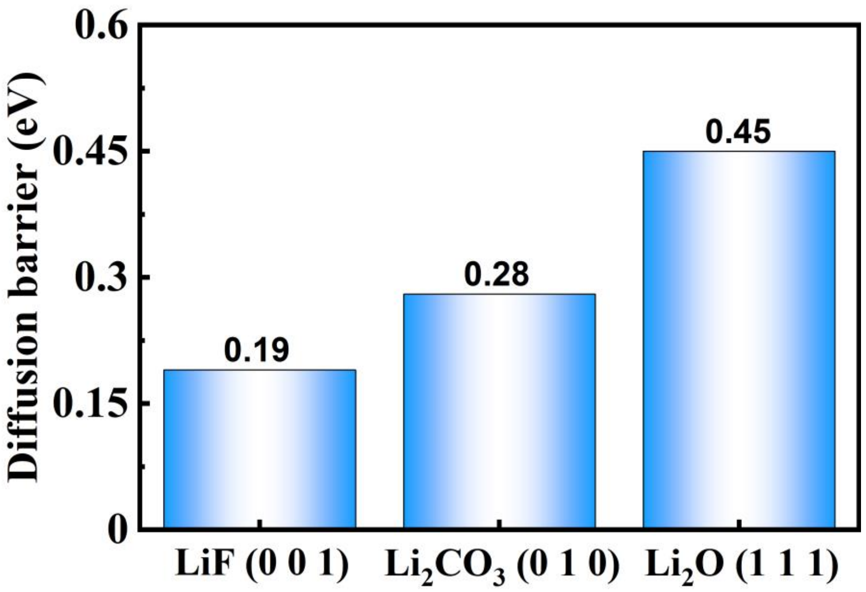

Supplementary Figure 40 | Diffusion barriers for Li^+ in LiF (0 0 1), Li_2CO_3 (0 1 0), and Li_2O (1 1 1). Li_2CO_3 and Li_2O are the main components of traditional SEI.

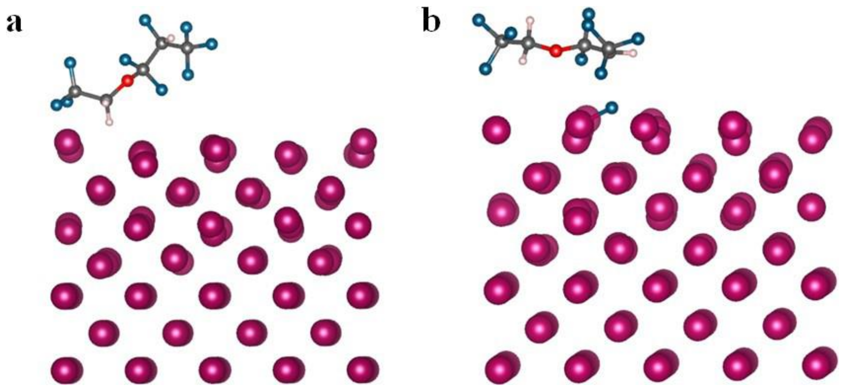

Supplementary Figure 41 | C-F bond breaking process of fluorine-rich THE on deposited Li (0 0 1) surface.

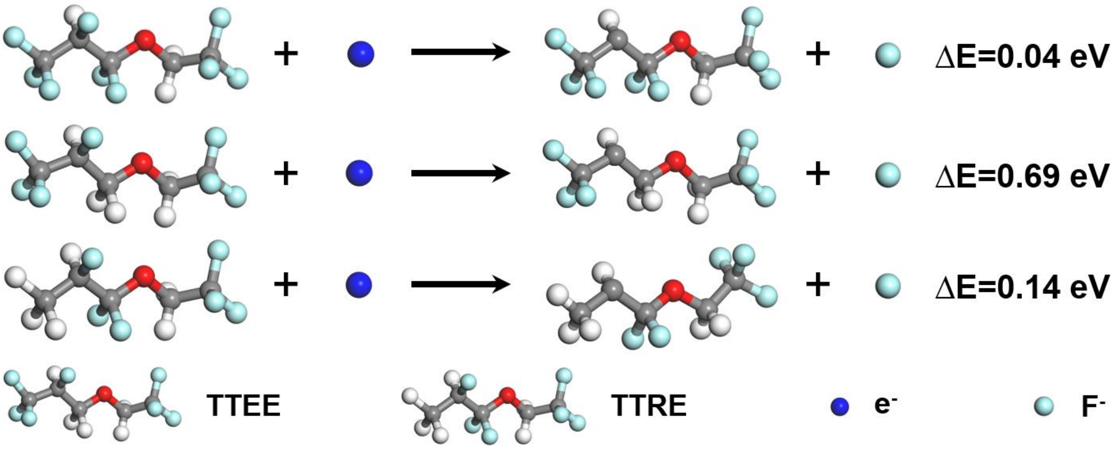

Supplementary Figure 42 | The breaking energy for the C-F bond cleavage of THE, TTEE (CF_2 is replaced with CH_2) and TTRE (CF_3 is replaced with CH_3).

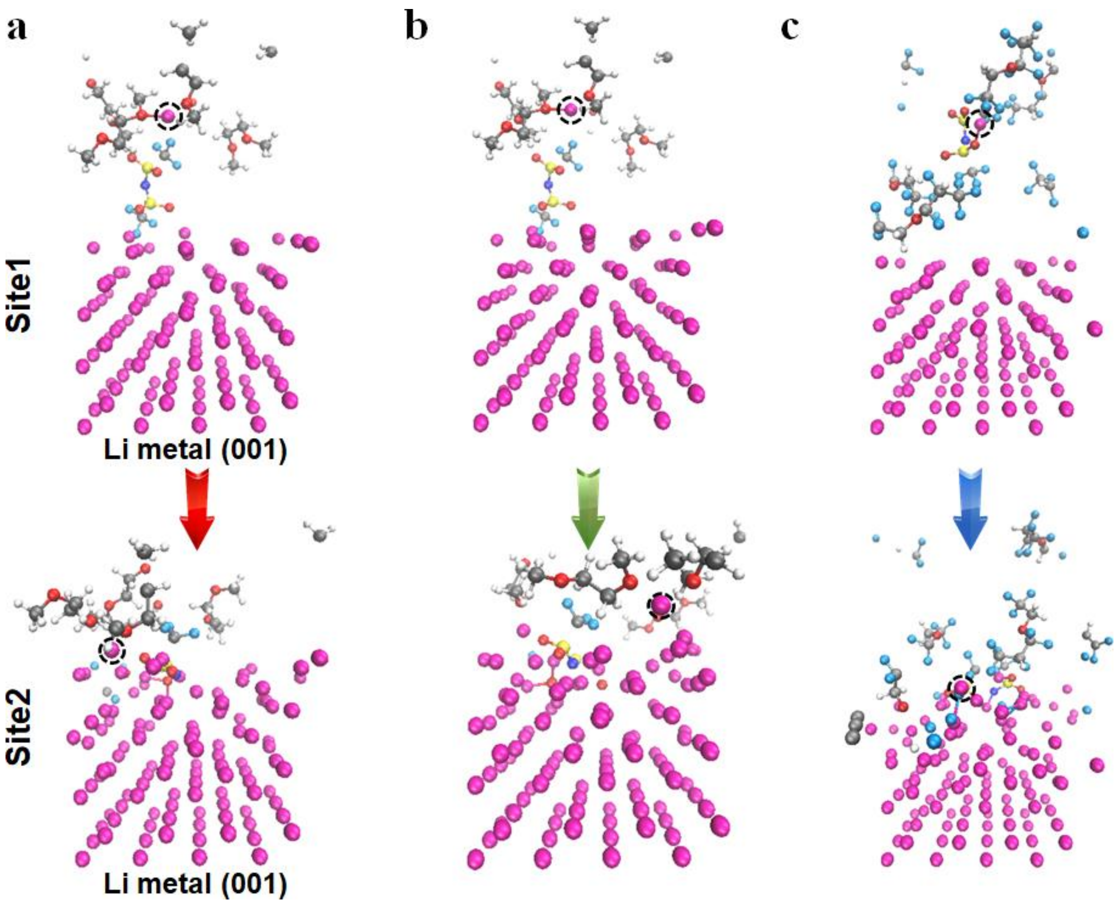

Supplementary Figure 43 | Initial and final configurations of (a) DME, (b) DOL, and (c) THE solvents at deposited Li (0 0 1) surface from AIMD calculations. Dashed circles indicate Li transfer reaction from solvents to the deposited Li (0 0 1) surface.

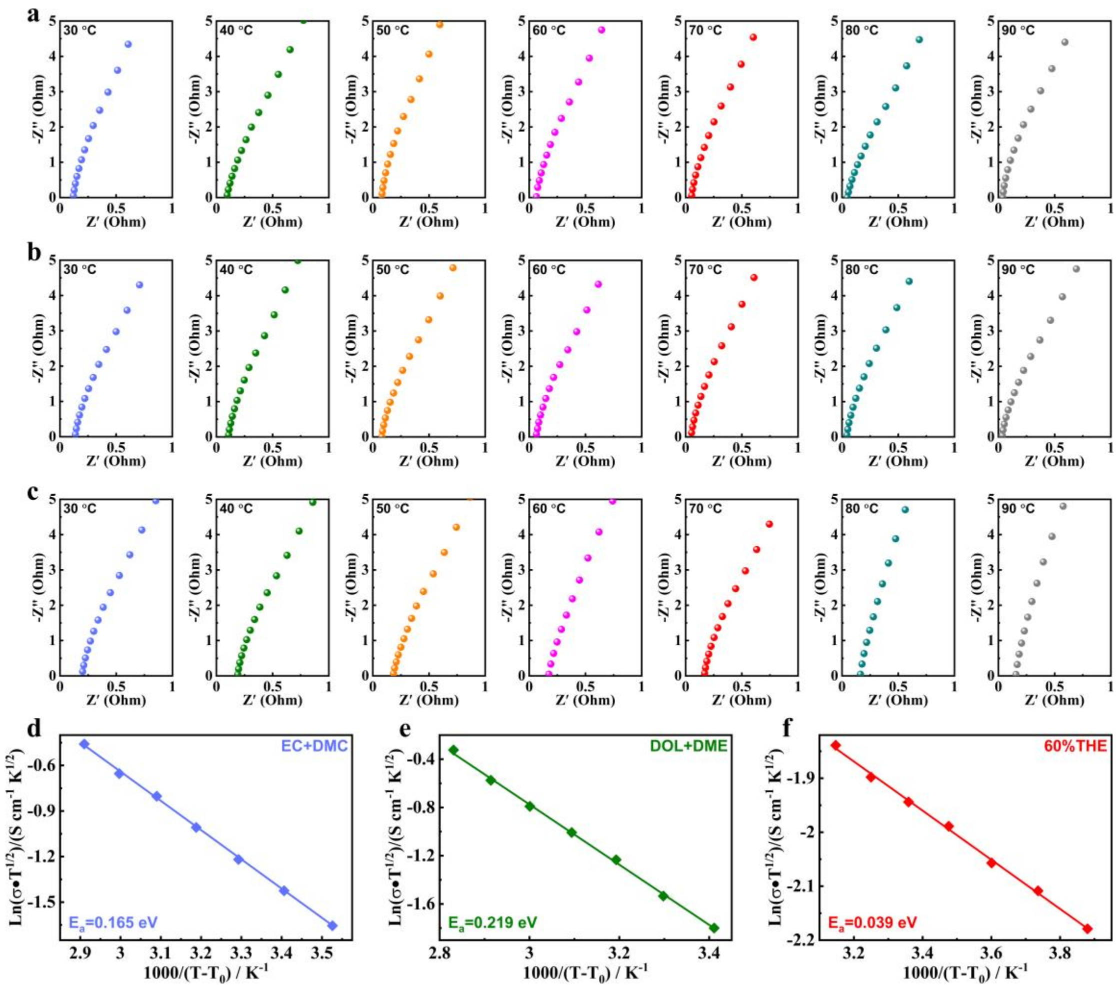

Supplementary Figure 44 | Nyquist plots for the impedances of the cells with (a) EC+DMC, (b) DOL+DME, and (c) 60%THE electrolytes at different temperatures. The activation energy values for (d) EC+DMC, (e) DOL+DME, and (f) 60%THE electrolytes. The three electrolytes were fitted by the following VTF empirical equation.

$$\sigma = \sigma_0 T^{-1/2} exp(-\frac{E_a}{R(T-T_0)}) \quad (7)$$

where E_a is the pseudo-activation energy, σ_0 is the pre-exponential factor, T_0 is the ideal glass transition temperature, and R is the gas constant.

Supplementary Table 1

Group	Key component THE (vt%)	DOL (vt%)	DME (vt%)	DMC (vt%)	EC (vt%)	FEC (vt%)	VC (vt%)	LiTFSI (mol/L)	$LiPF_6$ (mol/L)	Name in our work
E1	10	45	45	/	/	/	/	1	/	10%THE
E2	20	40	40	/	/	/	/	1	/	20%THE
E3	30	35	35	/	/	/	/	1	/	30%THE
E4	40	30	30	/	/	/	/	1	/	40%THE
E5	45	27.5	27.5	/	/	/	/	1	/	45%THE
E6	50	25	25	/	/	/	/	1	/	50%THE
E7	55	22.5	22.5	/	/	/	/	1	/	55%THE
**E8**	**60**	**20**	**20**	/	/	/	/	**1**	/	**60%THE**
E9	65	17.5	17.5	/	/	/	/	1	/	65%THE
E10	70	15	15	/	/	/	/	1	/	70%THE
E11	75	12.5	12.5	/	/	/	/	1	/	75%THE
E12	80	10	10	/	/	/	/	1	/	80%THE
R1	/	50	50	/	/	/	/	1	/	DOL+DME
R2	/	/	/	50	50	/	/	/	1	EC+DMC
R3	/	/	/	44	44	10	2	/	1	EC+DMC+10 %FEC+2%VC

E1-E12 are self-made electrolytes. Among them, the 60%THE electrolyte exhibits the best comprehensive electrochemical performance, as the key research object. R1, R2 and R3 are commercial electrolytes as references.

Supplementary Table 2

Rate (C)	60%THE initial capacity (mAh g^{-1})	DOL+DME initial capacity (mAh g^{-1})	60% THE retention (%)	DOL+DME retention (%)	Cycle number
0.1	166.8	166.5	99.9	98.7	100
1	153.1	130.3	99.9	85.6	100
10	93.8	63.7	94.1	7.1	5000
20	84.4	48.5	93.6	33.0	2000
40	81.3	31.8	99.4	44.2	2000
60	74.8	14.7	93.3	39.5	2000
80	45.8	12.5	71.8	37.6	2000
100	37.8	11.8	60.8	16.9	2000

Supplementary Table 3

Rate (C)	Cycle number	Retention (%)	Decay Per Cycle (%)	Ref. No
1	300	99.30	0.0023	8
1	1000	94.00	0.0060	9
1	1000	96.60	0.0034	10
1	800	93.30	0.0084	11
1	890	77.57	0.0252	12
2	500	92.20	0.0156	13
3	3350	80.00	0.0060	14
5	200	98.50	0.0075	15
5	500	91.00	0.0180	16
10	500	91.24	0.0175	17
10	700	91.70	0.0119	18
10	700	97.70	0.0033	19
10	950	91.40	0.0091	20
10	1000	89.00	0.0110	21
10	1000	84.00	0.0160	22
10	1000	92.23	0.0078	23
10	5000	60.00	0.0080	24
10	1000	98.40	0.0016	25
6	1000	82.50	0.0175	26

2	700	84.00	0.0229	27
5	1000	87.00	0.0130	28
2	1000	85.20	0.0148	29
5	1000	97.30	0.0027	30
5	4000	74.20	0.0065	31
5	1000	83.80	0.0162	32
**10**	**5000**	**94.14**	**0.0012**	**our work**

Supplementary Table 4

Rate (C)	Specific Capacity (mAh g^{-1})	Ref. No
2	~116	33
2	~88	34
2	~84	35
5	~102	36
8	~98	26
8	~65	37
10	~82	27
10	~111	28
**10**	**124**	**our work**

Supplementary Table 5

Rate (C)	25 °C 60%THE initial capacity (mAh g^{-1})	0 °C 60%THE initial capacity (mAh g^{-1})	60%THE initial capacity difference (mAh g^{-1})	25 °C DOL+DME initial capacity (mAh g^{-1})	0 °C DOL+DME initial capacity (mAh g^{-1})	DOL+DME initial capacity difference (mAh g^{-1})
1	153.2	151.7	1.5	130.8	122.8	8.0
5	124.1	120.4	3.7	94.2	82.4	11.8
10	108.7	103.8	4.9	67.5	61.3	6.2
20	95.1	92.4	2.7	45.8	38.8	7.0
40	81.7	79.8	1.9	31.5	33.6	-2.1
60	72.8	68.9	3.9	15.2	20.3	-5.1
80	49.2	46.7	2.5	10.1	15.8	-5.7
100	37.2	33.5	3.7	8.1	10.2	-2.1

Supplementary Table 6

Component	DOL+DME ion conductivity (S/cm)	60%THE ion conductivity (S/cm)
$LiFePO_4$ cathode	$\sim 10^{-4}$ [38]	$\sim 10^{-4}$ [38]
electrolyte/cathode interface	$\sim 4.5 \times 10^{-7}$ [39]	$\sim 4.5 \times 10^{-7}$ [39]
electrolyte	9.2	6.2
electrolyte/anode interface	1×10^{-9} [40]	1.9×10^{-7}
Graphite anode	$\sim 10^{-3}$ [41]	$\sim 10^{-3}$ [41]

Supplementary Table 7

Other cathode and anode	Ion conductivity (S/cm)
NCM532 cathode	1.7×10^{-3} [42]
NCM811 cathode	6.3×10^{-3} [42]
$LiCoO_2$ cathode	$\sim 10^{-6}$ [42]
$Li_4Ti_5O_{12}$ anode	$\sim 10^{-5}$ [43]

Reference:

[1] H. Zeng, H. Xiang, F. Jiang, Y. Liu, Y. Sun, X. Liang, Y. Feng, Y. Yu, *Adv. Energy Mater.* **2020**, *10*, 2001440.

[2] W.-L. Jorgensen, D.-S. Maxwell, J. TiradoRives, *J.Am.Chem.Soc.* **1996**, *118*, 11225.

[3] W. Damm, A. Frontera, J. TiradoRives, W.-L. Jorgensen, *J. Comput. Chem.* **1997**, *18*, 1955.

[4] L.-S. Dodda, I.-C. DeVaca, J. TiradoRives, W.-L. Jorgensen, *Nucleic Acids Res.* **2017**, *45*, W331.

[5] L.-S. Dodda, J.-Z. Vilseck, J. TiradoRives, W.-L. Jorgensen, *J. Phys. Chem. B* **2017**, *121*, 3864.

[6] J. Lopes, A. Padua, *J. Phys. Chem. B* **2004**, *108*, 16893.

[7] L. Martinez, R. Andrade, E.-G. Birgin, J.-M. Martinez, *J. Comput. Chem.* **2009**, *30*, 2157.

[8] X. Tian, Y. Zhou, G. Wu, P. Wang, J. Chen, *Electrochim. Acta* **2017**, *229*, 316.

[9] A. Shellikeri, S. Yturriaga, J. S. Zheng, W. Cao, M. Hagen, J. A. Read, T. R. Jow, J. P. Zheng, *J. Power Sources* **2018**, *392*, 285.

[10] Z. Zhang, Y. Zhao, S. Chen, D. Xie, D. Xao, X. Yao, P. Cui, X. Xu, *J. Mater. Chem. A* **2017**, *5*, 16984.

[11] G. Du, Y. Zhou, X. Tian, G. Wu, Y. Xi, S. Zhao, *Appl. Surf. Sci.* **2018**, *453*, 493.

[12] Y. Liu, Q. Liu, L. Xin, Y. Liu, F. Yang, E. A. Stach, J. Xie, *Nat. Energy* **2017**, *2*, 17083.

[13] J. Oh, J. Lee, Y. Jeon, S. Park, J. M. Kim, T. Hwang, Y. Piao, *ACS Sustainable Chem. Eng.* **2019**, *7*, 306.

[14] A. Kvasha, I. Urdampileta, I. Demeatza, M. Bengoechea, J. Blazquez, L. Yate, O. Miguel, H.-J. Grande, *Electrochim. Acta* **2016**, *215*, 238.

[15] Y. Li, Y. Zhang, J. Ma, L. Yang, X. Li, E. Zhao, S. Fan, G. Xu, S. Yang. C. Yang, *J. Electrochem. Soc.* **2019**, *166*, A410.

[16] L. Gao, Y. Jin, X. Liu, M. Xu, X. Lai, J. Shui, *Nanoscale* **2018**, *10*, 7676.

[17] C.-C. Yang, J.-R. Jiang, C. Karuppiah, J.-H. Jang, Z.-H. Wu, R. Jose, S. J. Lue, *J. Alloys Compd.* **2018**, *765*, 800.

[18] G. Longoni, J. K. Panda, L. Gagliani, R. Brescia, L. Manna, F. Bonaccorso, V. Pellegrini, *Nano Energy* **2018**, *51*, 656.

[19] J. Oh, J. Lee, T. Hwang, J. M. Kim, K.-D. Seoung, Y. Piao, *Electrochim. Acta* **2017**, *231*, 85.

[20] W.-B. Luo, S.-L. Chou, Y.-C. Zhai, H.-K. Liu, *J. Mater. Chem. A* **2014**, *2*, 4927.

[21] B. Wang, W. Al Abdulla, D. Wang, X. S. Zhao, *Energy Environ. Sci.* **2015**, *8*, 869.

[22] J. Hwang, K. C. Kong, W. Chang, E. Jo, K. Nam, J. Kim, *Nano Energy* **2017**, *36*, 398.

[23] Y. Zou, S. Chen, X .Yang, N. Ma, Y. Xia, D. Yang, S. Guo, *Adv. Energy Mater.* **2016**, *6*, 1601549.

[24] K. Yamamoto, D. Suemasa, K. Masuda, K. Aita, T. Endo, *ACS Appl. Mater.*

Interfaces **2018**, *10*, 6346.
[25] Y. Q. Qiao, W. L. Feng, J. Li, T. D. Shen, *Electrochim. Acta* **2017**, *232*, 323.
[26] Y. He, H. Li, S. Huo, Y. Chen, Y. Zhang, Y. Wang, W. Cai, D. Zeng, C. Li, H. Cheng, *J. Power Sources* **2021**, *490*, 229545.
[27] X. Song, Y. Zhang, Y. Ye, Z. Liu, F. Cheng, H. Li, *ACS. Appl. Energy Mater.* **2020**, *3*, 4906.
[28] Z. Wang, F. Zhang, Y. Sun, L. Zheng, Y. Shen, D. Fu, W. Li, A. Pan, L. Wang, J. Xu, J. Hu, X. Wu, *Adv. Energy Mater.* **2021**, *11*, 2003752.
[29] R. Zhang, Y. Li, L. Qiao, D. Li, J. Deng, J. Zhou, L. Xie, Y. Hou, T. Wang, W. Tian, J. Cao, F. Cheng, B. Yang, K. Liang, P. Chen, B. Kong, *Energy Storage Mater.* **2021**, *37*, 123.
[30] H. Zheng, Q. Zhang, Q. Chen, W. Xu, Q. Xie, Y. Cai, Y. Ma, Z. Qiao, Q. Luo, J. Lin, L. Wang, B. Qu, B. Sa, D.-L. Peng, *J. Mater. Chem. A* **2020**, *8*, 313.
[31] H. Wang, P. Hu, X. Liu, Y. Shen, L. Yuan, Z. Li, Y. Huang, *Adv. Sci.* **2021**, *8*, 2100684.
[32] Y. Liang, Y. Chen, X. Ke, Z. Zhang, W. Wu, G. Lin, Z. Zhou, Z. Shi, *J. Mater. Chem. A* **2020**, *8*, 18094.
[33] S. Fang, L. Qu, D. Luo, S. Shen, L. Yang, S. Hirano, *Rsc. Adv.* **2015**, *5*, 33897.
[34] Q. Ma, Z. Fang, P. Liu, J. Ma, X. Qi, W. Feng, J. Nie, Y.-S. Hu, H. Li, X. Huang, L. Chen, Z. Zhou, *Chemelectrochem.* **2016**, *3*, 531.
[35] X. Li, S. Li, Z. Zhang, J. Huang, L. Yang, S. Hirano, *J. Mater. Chem. A* **2016**, *4*, 13822.
[36] X. Liu, C. Shen, N. Gao, Q. Hou, F. Song, X. Tian, Y. He, J. Huang, Z. Fang, K. Xie, *Electrochim. Acta* **2018**, *289*, 422.
[37] V. Ramar, C. Pszolla, M. Rapp, M. Brock, L. Zinck, *J. Electrochem. Soc.* **2020**, *167*, 070521.
[38] P. Prosini, D. Zane, M. W. Pasquali, *Electrochim. Acta* **2001**, *46*, 3517.
[39] F. Zhang, C. Wang, D. Zhao, L. Yang, P. Wang, W. Li, B. Wang, S. Li, *Electrochim. Acta* **2020**, *337*, 135727.
[40] B. Han, D. Feng, S. Li, Z. Zhang, Y. Zou, M. Gu, H. Meng, C. Wang, K. Xu, Y. Zhao, H. Zeng, C. Wang, Y. Deng, *Nano. Lett.* **2020**, *20*, 4029.
[41] M. Yoshio, H. Wang, K. Fukuda, *Angew. Chem., Int. Ed. Engl.* **2003**, *42*, 4203.
[42] S. Wang, M. Yan, Y. Li, C. Vinado, J. Yang, *J. Power Sources* **2018**, *393,* 75.
[43] S. Kim, B. Na, *Korean Chem. Eng. Res.* **2017**, *55*, 430.
